# The synthesis and evaluation of triazolopyrimidines as anti-tubercular agents

**DOI:** 10.1016/j.bmc.2017.05.030

**Published:** 2017-08-01

**Authors:** Edison S. Zuniga, Aaron Korkegian, Steven Mullen, Erik J. Hembre, Paul L. Ornstein, Guillermo Cortez, Kallolmay Biswas, Naresh Kumar, Jeffrey Cramer, Thierry Masquelin, Philip A. Hipskind, Joshua Odingo, Tanya Parish

**Affiliations:** a TB Discovery Research, Infectious Disease Research Institute, 1616 Eastlake Avenue East, Seattle, WA 98102, USA; b Lilly Research Laboratories, Eli Lilly and Company, Indianapolis, IN 46285, USA; c Roosevelt University College of Pharmacy, Schaumburg, IL 60173, USA; d Jubilant Chemsys Limited, B-34, Sector 58, Noida 201301, India

**Keywords:** Tuberculosis, *Mycobacterium tuberculosis*, Anti-tubercular activity, Triazolopyrimidines

## Abstract

We identified a di-substituted triazolopyrimidine with anti-tubercular activity against *Mycobacterium tuberculosis*. Three segments of the scaffold were examined rationally to establish a structure-activity relationship with the goal of improving potency and maintaining good physicochemical properties. A number of compounds displayed sub-micromolar activity against *Mycobacterium tuberculosis* with no cytotoxicity against eukaryotic cells. Non-substituted aromatic rings at C5 and a two-carbon chain connecting a terminal aromatic at C7 were preferred features; the presence of NH at C7 and a lack of substituent at C2 were essential for potency. We identified compounds with acceptable metabolic stability in rodent and human liver microsomes. Our findings suggest that the easily-synthesized triazolopyrimidines are a promising class of potent anti-tubercular agents and warrant further investigation in our search for new drugs to fight tuberculosis.

## Graphical abstract

**Figure:**
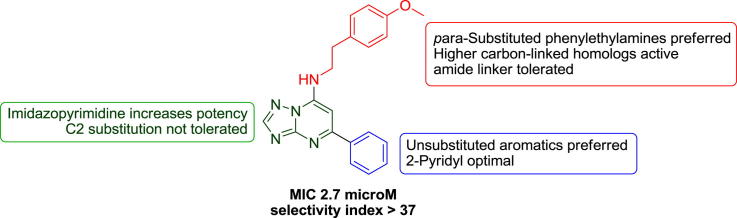

## 1 Introduction

Tuberculosis (TB) and its causative agent *Mycobacterium tuberculosis* present a serious threat to global health. There are over 1 million deaths each year and approximately 9 million new cases.^1^ Taken together with the estimate that a third of the world’s population is infected with *M. tuberculosis* and the existence of drug-resistant strains of *M. tuberculosis*, it is apparent there is a pressing need for new therapies. To address these needs, there has been an increased effort directed towards TB drug discovery in recent years and a pipeline of new anti-TB drug candidates has started to emerge. The search for new molecular scaffolds with potentially novel mechanisms of action remains a priority.

Triazolopyrimidines (TZPs) are a well-known scaffold in medicinal chemistry, and their utility is exemplified by the discovery and development of novel agents to fight a wide range of diseases. For example, TZPs possess anticancer activity,^2^ and have been used as phosphodiesterase inhibitors for diabetes treatment.^3^ Recently, the first natural TZP, essramycin, was isolated and found to possess antibacterial activity.^4^ A considerable effort has been made to develop TZPs with antimalarial activity.^5^, ^6^, ^7^ Transition metal-containing TZPs have antiproliferative activity against *Leishmania* and *Trypanosoma cruzi*, the protozoa that cause leishmaniasis and Chagas disease, respectively.^8^ In addition TZP acylsulfonamides with anti-mycobacterial activity target acetohydroxyacid synthase.^9^ Similar compounds were also identified in a phenotypic screening campaign against *Mycobacterium bovis* BCG.^10^

**Figure:**
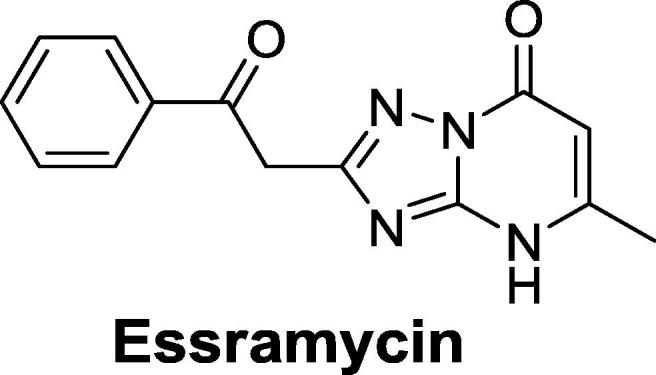

**Fig. 1:**
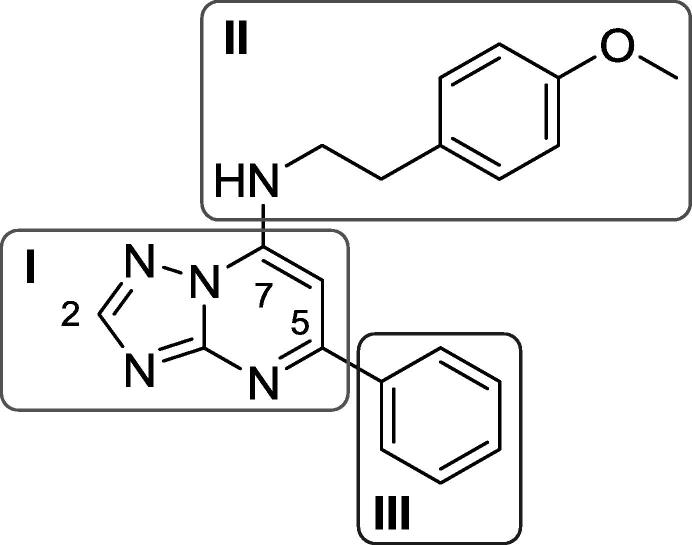
Triazolopyrimidine (TZP) **1**. Three segments are illustrated with boxes and numbered.

We identified a single TZP compound (**1**) from a whole-cell screen against *M. tuberculosis* which was active in liquid culture (Fig. 1). The compound had good activity against *M. tuberculosis* with a minimum inhibitory concentration (MIC) of 3.1 μM (Table 1). The compound was not cytotoxic, with an IC_50_ of >100 μM against the HepG2 cell line; the selectivity index (SI), defined as the ratio of cytotoxicity to MIC, was >32. Based on these data we initiated a structure-activity relationship (SAR) study around this singleton.

**Table 1:** Examining Aromatic Moieties at C5.

**Figure:**
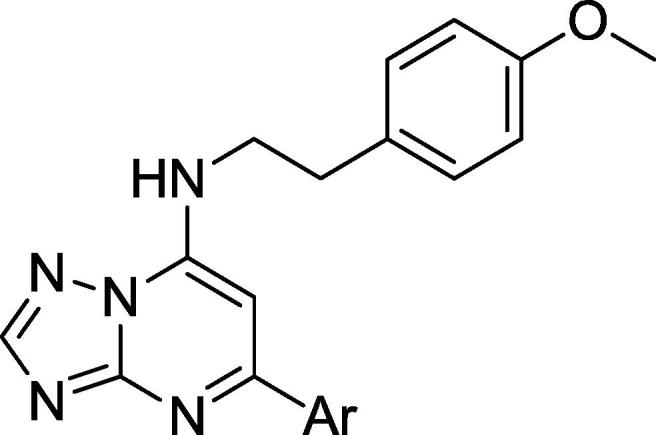

Cpd	Ar	MIC (μM)	IC_50_ (μM)	SI	Cpd	Ar	MIC (μM)	IC_50_ (μM)	SI
**1**		3.1 ± 1.3	>100	>32	**13**		4.4 ± 2.0	89	>21
**7**		>20	>100	**–**	**14**		3.0 ± 0.21	>100	>30
**8**		>20	87	**–**	**15**		>20	>100	–
**9**		>20	37	**–**	**16**		0.83 ± 0.26	>100	>130
**10**		>20	>100	**–**	**17**		>20	>100	–
**11**		>20	65	**–**	**18**		>20	69	–
**12**		>20	>100	**–**	**19**		>20	57	–

MIC_90_ is the minimum concentration required to inhibit growth of *M. tuberculosis* by 90%. MICs are the average ± standard deviation of two independent experiments. IC_50_ is the concentration required to reduce the viability of HepG2 cells by 50%. Selectivity index (SI) = IC_50_/MIC_90_.

Here, we present an exploratory study to understand the SAR of the TZP series. We identified key functionalities and features necessary for anti-tubercular activity. In general, TZP compounds lack cytotoxicity and display an encouraging metabolic stability profile. In addition, we demonstrated their bactericidal activity against non-replicating bacteria.

## 2 Results and discussion

Our SAR investigation began with the design and synthesis of novel analogs based on modifications of the core structure of compound **1**. We set out to explore modifications of the core by way of heteroatom replacement and the impact of chemical diversity at the C2, C5, and C7 positions.

### 2.1 Chemical synthesis

**Scheme 1:**
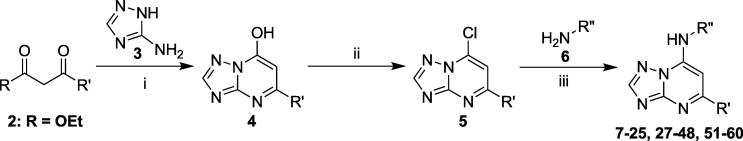
Synthesis of the triazolopyrimidine compounds. General conditions: (i) AcOH, 120 °C, 12–16 h; (ii) POCl_3_, 80 – 100 °C, 2 h; (iii) NMP, 80–100 °C or room temperature.

**Scheme 2:**
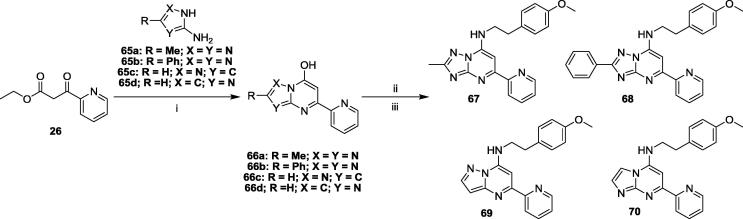
Synthesis of triazolopyrimidine compounds exploring core modifications. General conditions: (i) AcOH, 100–120 °C, 12–16 h; (ii) POCl_3_, 80–100 °C, 2 h; (iii) amine **6**, NMP, 80–100 °C.

The general synthetic routes are shown in Scheme 1, Scheme 2. We initially evaluated the SAR associated with modifications at the C5 position of the TZP ring system. Synthesis of analogs **7**–**25** proceeded through key 7-hydroxytriazolopyrimidine intermediates **4** (Scheme 1), which were obtained via the condensation of the 1,3-di-keto compound **2** with the 5-amino-4*H*-1,2,4-triazole **3**.^9^ Di-keto compounds were prepared by treating the appropriate substituted ethanone with diethyl carbonate in the presence of base. Chloro intermediates **5** were obtained upon treatment of **4** with phosphoryl chloride. Reaction of **5** with 4-methoxyphenethylamine gave C5 substituted triazolopyrimidines **7**–**25** in moderate to good yields. Condensation between 3-(dimethylamino)-1-phenyl-propane-1-one and **3** directly gave **27.**

Compound **5** served as a strategic intermediate from which a diverse set of C7 analogs could be prepared. Condensation between **5** (R′ = phenyl) and a variety of amines (**6**) gave C7 substituted triazolopyrimidines **27**–**48** with good yields. Similarly, reaction of **5** (R′ = 2-pyridinyl) with a variety of amines gave C7 substituted analogs **51**–**60**; the use of cyclic amines gave **62**–**64**. Compound **28** was obtained after treating 7-chloro-5-phenyl-[1,2,4]triazolo[1,5-*a*]pyrimidine (**5a**) with methanolic ammonia. Synthesis of amide analogs **49** and **50** proceeded through **28** via EDCI-HOBt mediated coupling with the appropriate substituted benzeneacetic acid. The alkylation of compound **1** using iodomethane afforded analog **61**.

As shown in Scheme 2, decoration of the C2 position of the TZP core proceeded through intermediate **66a-b**, obtained from the condensation between ethyl 2-pyridylcarbonylacetate (**2k**) and a 2-substituted 5-amino-1*H*-1,2,4-triazole (**65a-b**). Commercially available triazoles **65a** and **65b** were treated with **26** to give **66a** and **66b**, respectively. Subsequent chlorination followed by treatment with 4-methoxyphenylethylamine gave C2 substituted TZP analogs **67** and **68**. Heterocyclic core replacement analogs pyrazolopyrimidine **69** and imidazopyridine **70** were produced after manipulation of commercially available intermediates **65c-d**.

### 2.2 Structure-activity relationship (SAR) studies

We determined the activity of all the compounds synthesized against both *M. tuberculosis* and eukaryotic cells (HepG2 cell line); we determined the MIC_90_ against *M. tuberculosis*, defined as the concentration required to inhibit 90% growth in liquid medium, and the IC_50_ against HepG2 cells, defined as the concentration required to reduce HepG2 viability by 50%. Selectivity index (SI) was calculated as IC_50_/MIC_90_. We completed a systematic evaluation by modification of the TZP ring at the C5, C7, C2 and core positions.

We first explored substitution of C5 phenyl with *ortho*- or *para*-electron-donating groups, which resulted in a loss of activity (Table 1, compounds **7**–**10**). The incorporation of fluorine on the para- or *ortho*-positions (compounds **13** and **14**) of the C5 phenyl ring was tolerated and maintained good separation from cytotoxicity. This was not seen with chloride analogs **11** and **12** which had no activity. The strongly deactivating *para*-CF_3_ phenyl moiety (compound **15**) abrogated activity. Replacing the C5 phenyl with a polar 2-pyridyl gave compound **16** with slightly increased potency while having no effect on cytotoxicity, and provided a valuable point for further optimization. It is interesting to note that 3- or 4-pyridyl isomers at C5 (**17** and **18**) resulted in loss of activity (Table 1).

No advantage was gained by replacing the aromatic group at C5 with either a linear alkyl (**20, 21**) or a cyclohexyl group (**25**) and only small cyclic alkyl moieties such as the cyclopropyl (**22**)**,** cyclopentyl **24** and cyclobutyl (**23**) analogs had good anti-tubercular activity (Table 2).

**Table 2:** Replacement of Aromatic Groups with Alkyl Moieties at C5.

**Figure:**
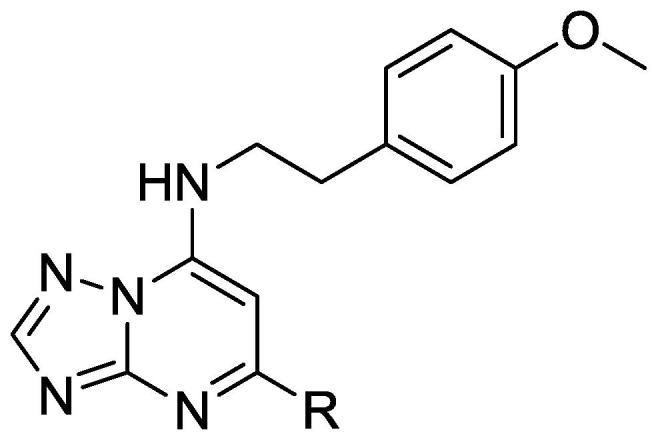

Cpd	R	MIC (μM)	IC_50_ (μM)	SI
**1**		3.1 ± 1.3	>100	>32
**20**		>20	>100	–
**21**		>20	>100	–
**22**		5.8 ± 1.6	>100	>17
**23**		2.9 ± 0.71	>100	>34
**24**		5.5 ± 0.07	95	17
**25**		>20	47	–

MIC_90_ is the minimum concentration required to inhibit growth of *M. tuberculosis* by 90%. MICs are the average ± standard deviation of two independent experiments. IC_50_ is the concentration required to reduce the viability of HepG2 cells by 50%. Selectivity index (SI) = IC_50_/MIC_90_.

We next explored modifications to the C7 position, while keeping a phenyl group at C5 (Table 3). Non-aromatic moieties at C7 (e.g. compounds **27**–**35**) did not show any anti-tubercular activity, except for the ethyl tethered cyclohexyl analog (**36**).

**Table 3:** Alkyl Chains at C7.

**Figure:**
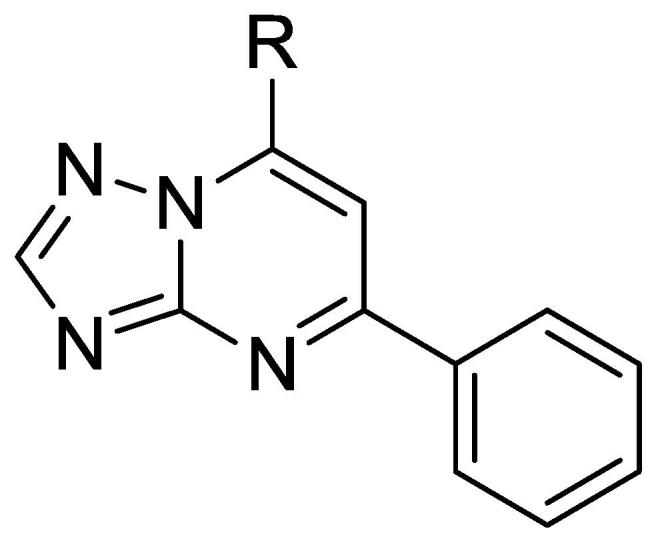

Cpd	R	MIC (μM)	IC_50_ (μM)	SI	Cpd	R	MIC (μM)	IC_50_ (μM)	SI
**27**		>20	>20	–	**32**		>20	98	–
**28**		>20	>20	–	**33**		>20	>100	–
**29**		>20	>20	–	**34**		>20	>100	–
**30**		>20	37	–	**35**		>20	57	–
**31**		>20	94	–	**36**		17 ± 2.1	>100	>6

MIC_90_ is the minimum concentration required to inhibit growth of *M. tuberculosis* by 90%. MICs are the average ± standard deviation of two independent experiments. IC_50_ is the concentration required to reduce the viability of HepG2 cells by 50%. Selectivity index (SI) = IC_50_/MIC_90_.

We next evaluated the effect of chain length at C7 (Table 4). Compounds **37** (with no tether), **38** (with a one carbon tether), and **40** (with a three carbon tether) were all inactive. A reduction in potency was observed for the unsubstituted terminal phenyl group as in compound **39** compared to the *para*-methoxy analog **1**. This pointed to the importance of *para*-substitution for activity and selectivity. Polar aromatics at the terminal end of the C7 alkyl chain were not tolerated as demonstrated by the inactive pyridyl analogs **41**–**43**. para-substituted analogs **44**–**48** were prepared, and the overall potency was rescued in all cases except for the *para*-fluoro analog **46**. C7 amide analogs (**49** and **50**) retained activity and were not cytotoxic indicating that properties of the aniline nitrogen at C7 can be modified with little penalty to activity.

**Table 4:** C7 Linker Modifications with C5 Phenyl.

**Figure:**
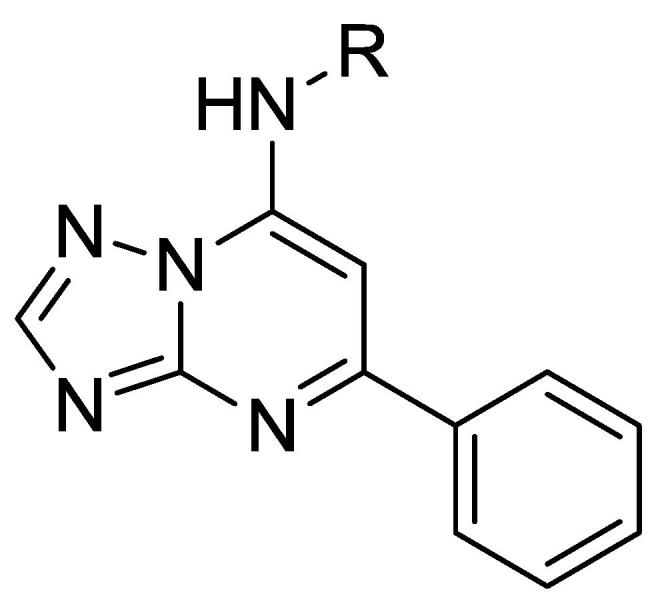

Cpd	R	MIC (μM)	IC_50_ (μM)	SI	Cpd	R	MIC (μM)	IC_50_ (μM)	SI
**37**		>20	49	–	**44**		1.4 ± 0.5	53	43
**38**		>20	43	–	**45**		0.74 ± 0.1	>100	>138
**39**		7.7 ± 0.7	27	3.5	**46**		>20	50	–
**40**		>20	8.7	–	**47**		2.7 ± 0.7	>100	>38
**41**		>20	>100	–	**48**		3.9 ± 1.1	>100	>27
**42**		>20	>100	–	**49**		1.5 ± 0.7	>100	>73
**43**		>20	>100	–	**50**		10 ± 1.1	>100	>10

MIC_90_ is the minimum concentration required to inhibit growth of *M. tuberculosis* by 90%. MICs are the average of two independent experiments. IC_50_ is the concentration required to reduce the viability of HepG2 cells by 50%. Selectivity index (SI) = IC_50_/MIC_90_.

Thus far we demonstrated that optimal anti-tubercular activity was found in the 2-pyridyl analog **16**. As with the C5 phenyl derivatives, we examined modifications to the terminal aromatic ring of the C7 side chain while keeping the 2-pyridyl at C5 constant (Table 5). A variety of *para*-substituted aromatic groups were tolerated at the terminal end of the C7 side chain resulting in analogs (**55**–**60**) with comparable or improved potency and good separation from cytotoxicity. It is worthy to note that analogs containing terminal pyridyl moieties on the C7 side chain (**52**–**54**) lost activity, as observed in the C5 phenyl series, demonstrating that polar residues are not tolerated in this region for either series.

**Table 5:** C7 Modifications with 2-Pyridyl at C5.

**Figure:**
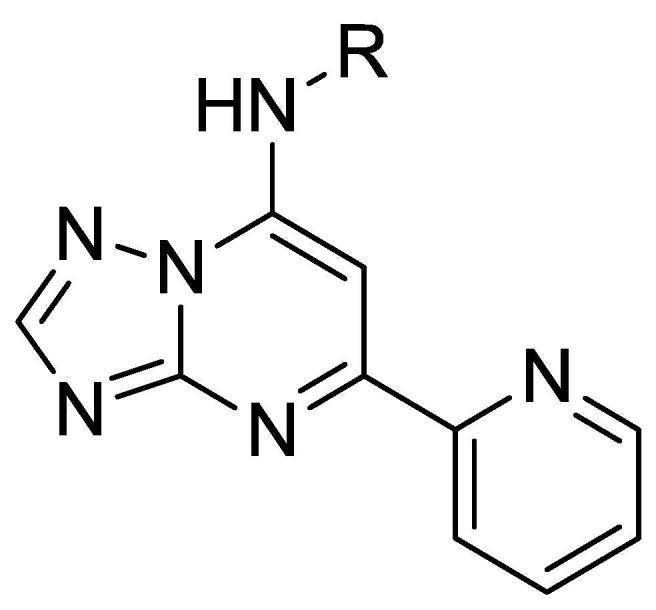

Cpd	R	MIC (μM)	IC_50_ (μM)	SI	Cpd	R	MIC (μM)	IC_50_ (μM)	SI
**51**		2.4 ± 1.3	>100	>46	**56**		0.2 ± 0.02	44	220
**52**		>20	>100	–	**57**		4.8 ± 1.3	81	17
**53**		>20	>100	–	**58**		0.98 ± 0.4	>100	>119
**54**		>20	>100	–	**59**		1.6 ± 1.1	>100	>72
**55**		0.52 ± 0.1	>100	>196	**60**		0.76 ± 0.25	>100	>135

MIC_90_ is the minimum concentration required to inhibit growth of *M. tuberculosis* by 90%. MICs are the average ± standard deviation of two independent experiments. IC_50_ is the concentration required to reduce the viability of HepG2 cells by 50%. Selectivity index (SI) = IC_50_/MIC_90_.

We investigated other SAR elements of the spacer at C7 (Table 6). The *N*-methylated analog **61** had a loss of activity, an indication that H-bonding may be important for the TZP compounds binding to their target (this could also be due to unfavorable steric interactions). Introducing rigidity on the amine tether as in pyrrolidine **62**, piperidine **63** and morpholino **64** was not tolerated. It has been previously reported that substitution on the C2 position of the TZP scaffold gave compounds with potent antimalarial activity and with good metabolic stability.^7^ Based on these findings and with the goal of identifying active and metabolically stable compounds, we prepared and tested the 2-methyl (**67**) and 2-phenyl (**68**) analogs. Analog **67** had comparable activity to our original hit, but analog **68** showed an MIC > 20 μM. Pyrazolopyrimidine^11^ and imidazopyridine^12^, ^13^ compounds have also been reported to possess potent anti-tubercular activity. Analog **69** with a pyrazolopyrimidine core demonstrated comparable activity to original hit **1**, but with increased cytotoxicity, whereas the imidazopyrimidine-based analog **70** had excellent anti-tubercular activity as well as good separation from cytotoxicity.

**Table 6:** Constraining C7 Side Chain and Core Modification.

Cpd	Structure	MIC (μM)	IC_50_ (μM)	SI	Cpd	Structure	MIC (μM)	IC_50_ (μM)	SI
**61**		>20	17	–	**67**		3.9 ± 0.21	>100	>26
**62**		>20	23	–	**68**		>20	>100	–
**63**		>20	16	–	**69**		2.5 ± 0.15	23	9
**64**		>20	50	–	**70**		0.22 ± 0.07	30	142

MIC_90_ is the minimum concentration required to inhibit growth of *M. tuberculosis* by 90%. MICs are the average ± standard deviation of two independent experiments. IC_50_ is the concentration required to reduce the viability of HepG2 cells by 50%. Selectivity index (SI) = IC_50_/MIC_90_.

### 2.3 Microsomal stability and *in vivo* pharmacokinetic (PK) studies

Based on *in silico* ADME predictions, three compounds were chosen to cover a range of cLogP values and were evaluated for their *in vitro* microsomal stability (Table 7). Rapid metabolism of compound **44** was observed, in rodent and human liver microsomes. The *para*-OCF_3_ analog **48** was also rapidly metabolized. The amide (**49**) had improved *in vitro* microsomal stability, with only 12% loss after 30 min in mouse microsomes, 22% loss in rat microsomes and 31% loss in human microsomes. The difference in stability for these three compounds is probably due to presence of more than one oxidatively-labile carbon in **44** and **48** (both have a two carbon linker), while **49** has only one such soft spot, although these have not been confirmed with metabolite identification studies.

**Table 7:** *In vitro* ADME and *in vivo* PK.

Cpd	% turnover by liver microsomes in 30 min			Mouse Fu,pl^a^	Predicted Fu,pl^b^	clogP^c^	PO AUC (nM*h)	PO Cmax (nM)	PO Tmax (h)	PO t1/2 (h)	IV clearance (mL/min/kg)
	Mouse	Rat	Human								
**44**	98.2	99.2	68.1	0.015	0.020	4.33	676	604	0.25	1.05	182
**48**	52.1	52.7	32.8	0.007	0.006	5.25	2750	958	0.75	2.49	33.5
**49**	12.5	21.8	30.6	ND	0.048	3.25	187	62.8	1.5	ND	248

a Fu,pl is the fraction unbound in plasma.

b Predicted value using a QSAR model built based on data generated for >3000 compounds measured internally (unpublished).

c clogP was predicted by Chemaxon model (www.chemaxon.com).

The oral exposure of the three compounds was evaluated in male mice by comparing exposures following oral (PO) and intravenous (IV) administration of 10 and 1 mg/kg doses of compounds, respectively (Table 7). Overall, the three compounds showed poor to moderate *in vivo* mouse PK properties. Compound **44** had an AUC = 676 nM∗h, and rapid IV clearance (182 mL/min/kg) as predicted by mouse microsomes. Compound **48** had more promising mouse PK with greater oral exposure (AUC 2750 nM * h), a longer half-life, and slower IV clearance (33.5 mL/min/kg). Surprisingly, despite showing good *in vitro* microsomal metabolic stability, amide **49** had the lowest oral exposure and fastest IV clearance rates *in vivo* compared to the two other compounds.

### 2.4 Activity Spectrum

Three compounds (**16**, **48** and **49**) were selected for testing against other organisms based on good activity in liquid and solid medium (MIC_99_ < 20 µM) against *M. tuberculosis* (Table 8). We tested against a non-pathogenic mycobacterial species (*Mycobacterium smegmatis*), *Escherichia coli* (Gram negative), *Pseudomonas aeruginosa* (Gram negative), *Bacillus subtilis* (Gram positive) and yeast (*Saccharomyces cerevisiae*) (Table 8). All three compounds had activity against *M. tuberculosis*, but no activity against any of the other species. Thus, the TZP compounds are selective for *M. tuberculosis*.

**Table 8:** Spectrum of activity.

MIC_99_ (μM)						
Cpd	*M. tuberculosis*	*M. smegmatis*	*E. coli*	*P. aeruginosa*	*S. cerevisiae*	*B. subtilis*
**16**	13	>100	>100	>100	>100	>100
**48**	1.6	>10	>10	>100	>10	>10
**49**	3.1	>10	>10	>10	>10	>10

MIC_99_ is the lowest concentration of compound, which yielded less than 1% growth.

### 2.5 Activity against replicating and non-replicating *M. tuberculosis*

**Fig. 2:**
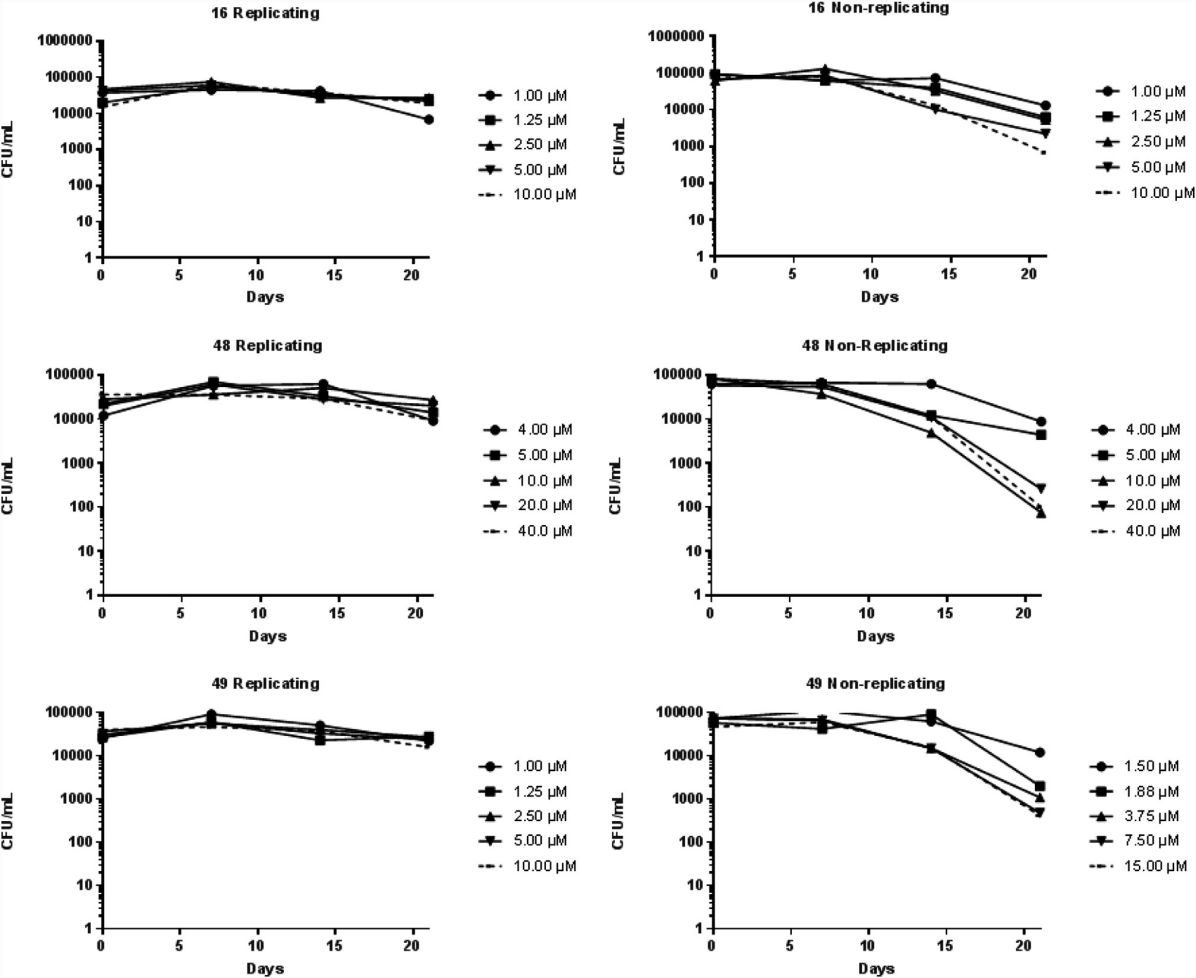
Kill kinetics against *M. tuberculosis* in liquid culture.

We also determined the effectiveness of the three compounds (**16**, **48** and **49**) in killing replicating and non-replicating *M. tuberculosis*. Compounds exhibited static activity against replicating bacteria, preventing growth, but no kill was noted over 21 days. In contrast, we did note killing against non-replicating bacteria (starvation conditions) for all three compounds, with a 2–3 log kill over 14–21 days (see Fig. 2).

## 3 Conclusion

We conducted a systematic exploration of the triazolopyrimidine scaffold for activity against *M. tuberculosis*. Overall, the compounds in this series show good activity and selectivity. Our initial explorations suggest that non-substituted aromatic rings at C5, and a two-carbon chain connecting a terminal aromatic at C7 are preferred features for potency against *M. tuberculosis* and separation from cytotoxicity. The presence of NH at C7 and a lack of substituent at C2 are essential for potency of the molecule. Heteroatom replacement or homologation of the scaffold is well tolerated in the series. We have identified compounds with improved metabolic stability in rodent and human liver microsomes, however, oral exposure and clearance remains an issue for this series. We feel that these issues can be improved through further SAR exploration. Thus, there is substantial promise in developing the TZP series that warrants further investigation as novel tools in our drug arsenal to combat tuberculosis.

## 4 Materials and methods

### 4.1 Determination of minimum inhibitory concentration (MIC)

MICs were determined against *M. tuberculosis* H37Rv as described in.^14^ Briefly, *M. tuberculosis* was grown in Middlebrook 7H9 medium containing 10% OADC (oleic acid, albumin, dextrose, catalase) supplement (Becton Dickinson) and 0.05% w/v Tween 80 (7H9-Tw-OADC) under aerobic conditions. Bacterial growth was measured by OD_590_ after 5 days of incubation at 37 °C. Curves were fitted using the Levenberg–Marquardt algorithm. MIC_90_ was defined as the minimum concentration required to inhibit growth of *M. tuberculosis* by 90%.

### 4.2 Determination of cytotoxicity (IC50)

Cytotoxicity was assessed using the HepG2 cell line under replicating conditions. HepG2 cells were grown in DMEM, High Glucose, GlutaMAX™ (Invitrogen), 10% fetal bovine serum (FBS), and 1X penicillin-streptomycin solution (100 U/mL). Cells were incubated with compounds for 2 days at 37 °C (final DMSO concentration of 1%), 5% CO2. CellTiter-Glo® Reagent (Promega) was added and relative luminescent units (RLU) measured to assess cell viaility. Inhibition curves were fitted using the Levenberg–Marquardt algorithm. IC_50_ is the concentration required to reduce cell viability after 2 days by 50%. Controls were 1% DMSO only (growth control) and staurosporine (positive control).

### 4.3 Determination of minimum inhibitory concentration on solid medium (MIC)

The serial proportion method was used to determine MIC_99_ on solid medium for a variety of representative species.^15^ *M. tuberculosis* H37Rv and *Mycobacterium smegmatis* mc^2^155 were grown in Middlebrook 7H9 medium + 10% v/v OADC (oleic acid, albumin, dextrose, catalase) supplement (Becton Dickinson) and on Middlebrook 7H10 medium + 10% v/v OADC. Plates were incubated at 37 °C for 4 weeks and 3–4 days respectively; *Escherichia coli* DH5α and *Staphylococcus aureus* RN4220 were grown on LB agar and incubated at 37 °C for 1 and 2 days respectively; *Pseudomonas aeruginosa* HER1018 (PAO1) was grown on tryptic soy agar and incubated at 37 °C for 1 day; *Bacillus subtilis* Marburg was grown in on nutrient agar and incubated at 28 °C for 3–4 days; *Saccharomyces cerevisiae* Y187 was grown on YPD agar plus 0.003% w/v adenine hemi-sulfate and incubated at 30 °C for 3–4 days. The MIC_99_ was the lowest concentration of compound, which yielded less than 1% growth relative to no-compound control.

### 4.4 Kill kinetics

To determine kill kinetics, replicating bacteria, a late log phase culture (OD_590_ 0.6–1.0) of *M. tuberculosis* was adjusted to an OD_590_ of 0.1 in 7H9-Tw-OADC; 50 µL was used to inoculate 5 mL 7H9-Tw-OADC containing compounds. For non-replicating conditions, bacteria were incubated at 37 °C for 14 days in PBS + 0.05% w/v Tyloxapol (PBS-Ty) at an OD_590_ of 0.1 and then compounds were added (final DMSO concentration of 2%). Cultures were incubated standing at 37 °C. Serial dilutions were plated on 7H10-OADC agar to determine CFU/mL.

### 4.5 Compound synthesis

#### 4.5.1 General methods

^1^H and ^13^C NMR spectral data were recorded in CDCl_3_ or DMSO-*d*_6_ on a 300 or 400 MHz Bruker NMR spectrometer. Column chromatography was conducted on silica gel (100–300 mesh). Reactions were monitored using thin-layer chromatography (TLC) on silica gel plates. HPLC analysis was conducted on an Agilent 1100 series LC system (Agilent ChemStation Rev.A.10.02; Phenomenex-Luna-C18, 4.8 mm × 150 mm, 5 μm, 1.0 mL/min, UV 254 nm, room temperature) with MeCN/H_2_O (0.05% TFA or HCOOH buffer) gradient elution. HPLCMSwas performed on a Gilson 321 HPLC with detection performed by a Gilson 170 DAD and a Finnigan AQA mass spectrometer operating in electrospray ionisation mode using a Phenomenex Gemini C18 150x4.6 mm column. Purity was determined using a Waters Acquity UPLC system equipped with a BEH C18 1.7 μm 2.1 × 100 mm column.

#### 4.5.2 General procedure for the synthesis of *N*-phenethyl-5-phenyl-[1,2,4]triazolo[1,5-*a*]pyrimidin-7-amines (**1**, **27**–**50**)

7-Chloro-5-phenyl-[1,2,4]triazolo[1,5-*a*]pyrimidine **4** (1.0 eq) was taken in NMP in a 100 mL round bottom flask under N_2_. To it was added amine precursor **6** (1.2 eq). The reaction mixture was heated at 90–120 °C or rt until complete by TLC analysis. The reaction mixture was then added to ice-cooled water and extracted with EtOAc. The organic layer was dried over anhydrous Na_2_SO_4_ and concentrated under reduced pressure. The crude was purified by flash chromatography on silica gel (100–200 mesh) using EtOAc-hexane or MeOH-DCM as eluent to afford compounds **1, 27**–**50.**

#### 4.5.3 General procedure for the synthesis of *N*-substituted-5-(pyridin-2-yl)-[1,2,4]triazolo[1,5-*a*]pyrimidin-7-amine (**16**, **51**–**60**)

7-Chloro-5-(pyridin-2-yl)-[1,2,4]triazolo[1,5-*a*]pyrimidine **5k** (1.0 eq) was taken in NMP in a 100 mL round bottom flask under N_2_. To it was added amine precursor (1.2 eq). The reaction mixture was heated at 90–100 °C or rt until complete by TLC analysis. The reaction mixture was then added to ice-cooled water and extracted with EtOAc. The organic layer was dried over anhydrous Na_2_SO_4_ and concentrated under reduced pressure. The crude was purified by flash chromatography on silica gel (100–200 mesh) using EtOAc-hexane or MeOH-DCM as eluent to afford compounds **16, 51**–**60.**

#### 4.5.4 *N*-(4-methoxyphenethyl)-5-phenyl-[1,2,4]triazolo[1,5-*a*]pyrimidin-7-amine (**1**)

7-chloro-5-phenyl-[1,2,4]triazolo[1,5-*a*]pyrimidine **5** (500 mg, 2.2 mmol) was taken in NMP (5 mL) in a 50 mL round bottom flask under N_2_. To it was added 2-(4-methoxyphenyl)ethan-1-amine (365 mg, 2.4 mmol). The reaction mixture was heated at 100 °C and monitored by TLC analysis (EtOAc, 100%) until completion. The reaction mixture was then poured into ice water (50 g) and extracted with EtOAc (2 × 50 mL). The combined organic layer was dried over anhydrous Na_2_SO_4_ and concentrated under reduced pressure. The crude was purified by flash chromatography on silica gel (100–200 mesh) using 30% EtOAc-hexane as eluent to afford **1** as a white solid (300 mg, 40%). M.P. 163–164 °C. ^1^H NMR (400 MHz, DMSO-*d*_6_): *δ* 8.46 (s, 1H), 8.34 (bs, 1H), 8.15–8.17 (m, 2H), 7.52–7.53 (m, 3H), 7.23 (d, *J* = 8.3 Hz, 2H), 6.84 (d, *J* = 7.2 Hz, 3H), 3.73–3.76 (m, 2H), 3.66 (s, 3H), 2.92–2.96 (m, 2H); ^13^C NMR (100 MHz, DMSO-*d*_6_): *δ* 160.2, 157.8, 155.5, 154.7, 147.9, 137.6, 130.7, 130.2, 129.9, 128.5, 127.4, 113.7, 84.8, 54.9, 43.2, 33.7. LCMS (ESI) *m*/*z* 346.45.

#### 4.5.5 Ethyl 3-(2-methoxyphenyl)-3-oxopropanoate (**2c**)

Sodium hydride (480 mg, 20.0 mmmol) was taken in dry THF (18 mL) in a 100 mL round bottom flask under N_2_ and cooled it down to 0 °C. To it was added a solution of ethyl 3-(2-methoxyphenyl)-3-oxopropanoate (1.0 g, 6.7 mmol) in THF (2 mL). The reaction mixture was stirred at rt for 30 min followed by the addition of diethyl carbonate (3.2 mL, 26.8 mmol). The reaction mixture was then stirred at rt for 14 h. Ice-cooled water was added dropwise to quench the reaction. It was extracted with EtOAc (3 × 75 mL). The combined organic layer was dried over anhydrous Na_2_SO_4_ and concentrated under reduced pressure. The crude was further purified by flash chromatography on silica gel (60–120 mesh) using 10% hexane-EtOAc as eluent to afford **2c** as a colourless liquid (1.3 g, 88%). LC-MS (ESI) *m*/*z* 223.32 [M+H^+^]; 97% (purity).

#### 4.5.6 Ethyl 3-oxo-3-(*p*-tolyl)propanoate (**2d**)

Sodium hydride (2.68 g, 112 mmmol) was taken in dry DMF (25 mL) in a 250 mL round bottom flask under N_2_ and cooled it down to 0 °C. To it was added a solution of 1-(*p*-tolyl)ethan-1-one (5.0 g, 37.3 mmol) in DMF (5 mL). The reaction mixture was stirred at rt for 30 min followed by the addition of diethyl carbonate (18 mL, 149.2 mmol). The reaction mixture was then stirred at rt for 16 h. Ice-cooled water was added dropwise to quench the reaction. It was extracted with EtOAc (3 × 100 mL). The combined organic layer was dried over anhydrous Na_2_SO_4_ and concentrated under reduced pressure. The crude was further purified by flash chromatography on silica gel (60–120 mesh) using 10% hexane-EtOAc as eluent to afford **2d** as a colourless liquid (6.2 g, 81%). LCMS (ESI) *m*/*z* 207.32 [M+H^+^]; 84.88% (purity).

#### 4.5.7 Ethyl 3-(4-ethylphenyl)-3-oxopropanoate (**2e**)

Sodium hydride (161 mg, 6.74 mmmol) was taken in dry THF (8 mL) in a 100 mL round bottom flask under N_2_ and cooled it down to 0 °C. To it was added a solution of 1-(4-ethylphenyl)ethan-1-one (500 mg, 3.37 mmol) in THF (2 mL). The reaction mixture was stirred at rt for 30 min followed by the addition of diethyl carbonate (1.6 mL, 13.5 mmol). The reaction mixture was then stirred at rt for 12 h. Ice-cooled water was added dropwise to quench the reaction. It was extracted with EtOAc (3 × 50 mL). The combined organic layer was dried over anhydrous Na_2_SO_4_ and concentrated under reduced pressure. The crude was triturated with Et_2_O afford **2e** as a brown solid (500 mg, 67%). LCMS(ESI) *m*/*z* 221.29 [M+H^+^]; 50% (purity).

#### 4.5.8 Ethyl 3-(2-fluorophenyl)-3-oxopropanoate (**2i**)

Sodium hydride (1.74 g, 72.4 mmmol) was taken in dry THF (30 mL) in a 100 mL round bottom flask under N_2_ and cooled it down to 0 °C. To it was added a solution of 1-(2-fluorophenyl)ethan-1-one (5.0 g, 36.2 mmol) in THF (5 mL). The reaction mixture was stirred at rt for 30 min followed by the addition of diethyl carbonate (17.5 mL, 144.8 mmol). The reaction mixture was then stirred at rt for 12 h. Ice-cooled water was added dropwise to quench the reaction. It was extracted with EtOAc (3 × 75 mL). The combined organic layer was dried over anhydrous Na_2_SO_4_ and concentrated under reduced pressure. The crude was triturated with Et_2_O afford **2i** as a brown liquid (4.0 g, 52%). MS (ESI) *m*/*z* 211.17 [M+H^+^].

#### 4.5.9 Ethyl 3-oxo-3-(4-(trifluoromethyl)phenyl)propanoate (**2j**)

Sodium hydride (2.55 g, 106.4 mmmol) was taken in dry THF (40 mL) in a 250 mL round bottom flask under N_2_ and cooled it down to 0 °C. To it was added a solution of 1-(4-(trifluoromethyl)phenyl)ethan-1-one (10 g, 53.2 mmol) in THF (10 mL). The reaction mixture was stirred at rt for 30 min followed by the addition of diethyl carbonate (25.8 mL, 212.8 mmol). The reaction mixture was then stirred at rt for 14 h. Ice-cooled water was added dropwise to quench the reaction. It was extracted with EtOAc (3 × 50 mL). The combined organic layer was dried over anhydrous Na_2_SO_4_ and concentrated under reduced pressure. The crude was triturated with Et_2_O afford **2j** as a brown solid (10.0 g, 72%). LCMS(ESI) *m*/*z* 259.14 [M−H^+^]; 61% (purity).

#### 4.5.10 Ethyl 3-oxo-3-(pyridin-3-yl)propanoate (**2l**)

Sodium hydride (2.9 g, 124 mmmol) was taken in dry THF (45 mL) in a 250 mL round bottom flask under N_2_ and cooled it down to 0 °C. To it was added a solution of 1-(pyridin-3-yl)ethan-1-one (5.0 g, 41.2 mmol) in THF (5 mL). The reaction mixture was stirred at rt for 30 min followed by the addition of diethyl carbonate (20 mL, 165.0 mmol). The reaction mixture was then stirred at rt for 14 h. Ice-cooled water was added dropwise to quench the reaction. It was extracted with EtOAc (3 × 100 mL). The combined organic layer was dried over anhydrous Na_2_SO_4_ and concentrated under reduced pressure. The crude was further purified by flash chromatography on silica gel (60–120 mesh) using 10% hexane-EtOAc as eluent to afford **2l** as a colourless liquid (4.1 g, 52%). LCMS(ESI) *m*/*z* 194.25 [M+H^+^]; 88% (purity).

#### 4.5.11 Ethyl 3-cyclobutyl-3-oxopropanoate (**2r**)

Sodium hydride (2.44 g, 101.9 mmmol) was taken in dry THF (55 mL) in a 250 mL round bottom flask under N_2_ and cooled it down to 0 °C. To it was added a solution of 1-cyclobutylethan-1-one (5.0 g, 50.9 mmol) in THF (5 mL). The reaction mixture was stirred at rt for 30 min followed by the addition of diethyl carbonate (24.7 mL, 203.6 mmol). The reaction mixture was then stirred at rt for 14 h. Ice-cooled water was added dropwise to quench the reaction. It was extracted with EtOAc (3 × 200 mL). The combined organic layer was dried over anhydrous Na_2_SO_4_ and concentrated under reduced pressure. The crude was further purified by flash chromatography on silica gel (60–120 mesh) using 10% hexane-EtOAc as eluent to afford **2r** as a colourless semi-solid (2.6 g, 30%). LCMS(ESI) *m*/*z* 169.07 [M−H^+^]; 80% (purity).

#### 4.5.12 Synthesis of 5-phenyl-[1,2,4]triazolo[1,5-*a*]pyrimidin-7-ol (**4**)

Ethyl 3-oxo-3-phenylpropanoate **2** (10.0 g, 52.1 mmol) was taken in AcOH (50 mL) in a 250 mL round bottom flask under N_2_. To it was added 1*H*-1,2,4-triazol-5-amine **3** (0.4 mL, 62.5 mmol). The reaction mixture was heated at 120 °C for 14 h. The reaction mixture was then evaporated to dryness using toluene as an azeotropic solvent and triturated with diethyl ether. This was finally dried under high vaccum which affored a white solid (10.5 g, 95%). This was then used in the next step without any further purification. LCMS(ESI) *m*/*z* 213.09 [M+H^+^]; 59.54% (purity).

#### 4.5.13 5-Phenyl-[1,2,4]triazolo[1,5-*a*]pyrimidin-7-ol (**4a**)

Ethyl 3-oxo-3-phenylpropanoate **2** (10.0 g, 52.1 mmol) was taken in AcOH (50 mL) in a 250 mL round bottom flask under N_2_. To it was added 1*H*-1,2,4-triazol-5-amine (0.4 mL, 62.5 mmol). The reaction mixture was heated at 120 °C for 14 h. The reaction mixture was then evaporated to dryness using toluene as an azeotropic solvent and triturated with diethyl ether. This was finally dried under high vacuum which afforded a white solid (10.5 g, 95%). This was then used in the next step without any further purification. LCMS(ESI) *m*/*z* 213.09 [M+H^+^]; 59.54% (purity).

#### 4.5.14 5-(4-Methoxyphenyl)-[1,2,4]triazolo[1,5-*a*]pyrimidin-7-ol (**4b**)

Ethyl 3-(4-methoxyphenyl)-3-oxopropanoate (1.0 g, 4.5 mmol) was taken in AcOH (5 mL) in a 50 mL round bottom flask under N_2_. To it was added 1*H*-1,2,4-triazol-5-amine (416 mg, 4.9 mmol). The reaction mixture was heated at 120 °C for 12 h. The reaction mixture was then evaporated to dryness using toluene as an azeotropic solvent and triturated with diethyl ether. This was finally dried under high vacuum which afforded **4b** as a brown solid (510 mg, 47%). This was then used in the next step without any further purification. LCMS(ESI) *m*/*z* 243.10 [M+H]^+^; 80.54% (purity).

#### 4.5.15 5-(2-Methoxyphenyl)-[1,2,4]triazolo[1,5-*a*]pyrimidin-7-ol (**4c**)

Ethyl 3-(2-methoxyphenyl)-3-oxopropanoate **2c** (1.5 g, 6.7 mmol) was taken in AcOH (15 mL) in a 100 mL round bottom flask under N_2_. To it was added 1*H*-1,2,4-triazol-5-amine **2** (681 mg, 8.1 mmol). The reaction mixture was heated at 120 °C for 14 h. The reaction mixture was then evaporated to dryness using toluene as an azeotropic solvent and triturated with diethyl ether. This was finally dried under high vacuum which afforded **4c** as an off-white solid (1.2 g, crude). This was then used in the next step without any further purification. LCMS(ESI) *m*/*z* 243.11 [M+H^+^]; 95.10% (purity).

#### 4.5.16 5-(*p*-Tolyl)-[1,2,4]triazolo[1,5-*a*]pyrimidin-7-ol (**4d**)

Ethyl 3-oxo-3-(*p*-tolyl)propanoate **2d** (6.0 g, 29.1 mmol) was taken in AcOH (40 mL) in a 250 mL round bottom flask under N_2_. To it was added 1*H*-1,2,4-triazol-5-amine (2.9 g, 34.9 mmol). The reaction mixture was heated at 120 °C for 14 h. The reaction mixture was then evaporated to dryness using toluene as an azeotropic solvent and triturated with diethyl ether. This was finally dried under high vacuum which afforded **4d** as a white solid (1.8 g, 27%). This was then used in the next step without any further purification. LCMS(ESI) *m*/*z* 227.22 [M+H^+^]; 75.24% (purity).

#### 4.5.17 5-(4-*E*thylphenyl)-[1,2,4]triazolo[1,5-*a*]pyrimidin-7-ol (**4e**)

Ethyl 3-(4-ethylphenyl)-3-oxopropanoate **2e** (500 mg, 2.27 mmol) was taken in AcOH (6 mL) in a 50 mL round bottom flask under N_2_. To it was added 1*H*-1,2,4-triazol-5-amine (286 mg, 3.4 mmol). The reaction mixture was heated at 115 °C for 16 h. The reaction mixture was then evaporated to dryness using toluene as an azeotropic solvent and triturated with diethyl ether. This was finally dried under high vacuum which afforded **4e** as a brown solid (400 mg, 73%). This was then used in the next step without any further purification. LCMS(ESI) *m*/*z* 239.20 [M−H^+^]; 45% (purity).

#### 4.5.18 5-(4-Chlorophenyl)-[1,2,4]triazolo[1,5-*a*]pyrimidin-7-ol (**4f**)

Ethyl 3-(4-chlorophenyl)-3-oxopropanoate (1.0 g, 4.4 mmol) was taken in AcOH (5 mL) in a 50 mL round bottom flask under N_2_. To it was added 1*H*-1,2,4-triazol-5-amine (406 mg, 4.8 mmol). The reaction mixture was heated at 120 °C for 16 h. The reaction mixture was then evaporated to dryness using toluene as an azeotropic solvent and triturated with diethyl ether. This was finally dried under high vacuum which afforded **4f** as a white solid (560 mg, 51%). This was then used in the next step without any further purification. LCMS(ESI) *m*/*z* 247.15 [M+H^+^]; 98.87% (purity).

#### 4.5.19 5-(4-Fluorophenyl)-[1,2,4]triazolo[1,5-*a*]pyrimidin-7-ol (**4h**)

Ethyl 3-(4-fluorophenyl)-3-oxopropanoate (2.0 g, 9.5 mmol) was taken in AcOH (10 mL) in a 50 mL round bottom flask under N_2_. To it was added 1*H*-1,2,4-triazol-5-amine **2** (958 mg, 11.4 mmol). The reaction mixture was heated at 120 °C for 12 h. The reaction mixture was then evaporated to dryness using toluene as an azeotropic solvent and triturated with diethyl ether. This was finally dried under high vacuum which afforded **4h** as a yellow solid (1.1 g, 50%). This was then used in the next step without any further purification. LCMS(ESI) *m*/*z* 231.25 [M+H^+^]; 79.73% (purity).

#### 4.5.20 5-(2-Fluorophenyl)-[1,2,4]triazolo[1,5-*a*]pyrimidin-7-ol (**4i**)

Ethyl 3-(2-fluorophenyl)-3-oxopropanoate **2i** (4.0 g, 19.0 mmol) was taken in AcOH (20 mL) in a 100 mL round bottom flask under N_2_. To it was added 1*H*-1,2,4-triazol-5-amine (1.92 g, 22.8 mmol). The reaction mixture was heated at 115 °C for 12 h. The reaction mixture was then evaporated to dryness using toluene as an azeotropic solvent and triturated with diethyl ether. This was finally dried under high vacuum which afforded **4i** as a yellow solid (800 mg, 18%). This was then used in the next step without any further purification. LCMS(ESI) *m*/*z* 231.06 [M+H^+^]; 54% (purity).

#### 4.5.21 5-(4-(Trifluoromethyl)phenyl)-[1,2,4]triazolo[1,5-*a*]pyrimidin-7-ol (**4j**)

Ethyl 3-oxo-3-(4-(trifluoromethyl)phenyl)propanoate **2j** (10.0 g, 38.5 mmol) was taken in AcOH (50 mL) in a 250 mL round bottom flask under N_2_. To it was added 1*H*-1,2,4-triazol-5-amine (4.85 g, 57.7 mmol). The reaction mixture was heated at 115 °C for 16 h. The reaction mixture was then evaporated to dryness using toluene as an azeotropic solvent and triturated with diethyl ether. This was finally dried under high vacuum which afforded **4j** as a yellow solid (6.0 g, 56%). This was then used in the next step without any further purification. LCMS(ESI) *m*/*z* 279.43 [M−H^+^]; 78% (purity).

#### 4.5.22 5-(Pyridin-2-yl)-[1,2,4]triazolo[1,5-*a*]pyrimidin-7-ol (**4k**)

Ethyl 3-oxo-3-(pyridin-2-yl)propanoate **2k** (8.0 g, 41.4 mmol) was taken in AcOH (50 mL) in a 250 mL round bottom flask under N_2_. To it was added 1*H*-1,2,4-triazol-5-amine (4.2 g, 49.7 mmol). The reaction mixture was heated at 120 °C for 16 h. The reaction mixture was then evaporated to dryness using toluene as an azeotropic solvent and triturated with diethyl ether. This was finally dried under high vacuum which afforded **4k** as a deep brown solid (4.5 g, 51%). This was then used in the next step without any further purification. MS (ESI) *m*/*z* 212.2 [M−H^+^].

#### 4.5.23 5-(Pyridin-3-yl)-[1,2,4]triazolo[1,5-*a*]pyrimidin-7-ol (**4l**)

Ethyl 3-oxo-3-(pyridin-3-yl)propanoate **2l** (4.0 g, 20.7 mmol) was taken in AcOH (40 mL) in a 250 mL round bottom flask under N_2_. To it was added 1*H*-1,2,4-triazol-5-amine (2.08 g, 24.8 mmol). The reaction mixture was heated at 120 °C for 14 h. The reaction mixture was then evaporated to dryness using toluene as an azeotropic solvent and triturated with diethyl ether. This was finally dried under high vacuum which afforded **4l** as a white solid (2.1 g, 48%). This was then used in the next step without any further purification. LCMS(ESI) *m*/*z* 214.13 [M+H^+^]; 62% (purity).

#### 4.5.24 5-(Pyridin-4-yl)-[1,2,4]triazolo[1,5-*a*]pyrimidin-7-ol (**4m**)

Ethyl 3-oxo-3-(pyridin-4-yl)propanoate (2.0 g, 10.4 mmol) was taken in AcOH (10 mL) in a 50 mL round bottom flask under N_2_. To it was added 1*H*-1,2,4-triazol-5-amine (1.04 g, 12.4 mmol). The reaction mixture was heated at 110 °C for 16 h. The reaction mixture was then evaporated to dryness using toluene as an azeotropic solvent and triturated with diethyl ether. This was finally dried under high vacuum which afforded **4m** as a yellow solid (1.2 g, 54%). This was then used in the next step without any further purification. LCMS(ESI) *m*/*z* 214.19 [M+H^+^]; 56% (purity).

#### 4.5.25 5-Benzyl-[1,2,4]triazolo[1,5-*a*]pyrimidin-7-ol (**4n**)

Ethyl 3-oxo-4-phenylbutanoate (1.5 g, 7.2 mmol) was taken in AcOH (8 mL) in a 50 mL round bottom flask under N_2_. To it was added 1*H*-1,2,4-triazol-5-amine (733 mg, 8.7 mmol). The reaction mixture was heated at 115 °C for 16 h. The reaction mixture was then evaporated to dryness using toluene as an azeotropic solvent and triturated with diethyl ether. This was finally dried under high vacuum which afforded **4n** as a white solid (910 mg, 55%). This was then used in the next step without any further purification.

#### 4.5.26 5-Methyl-[1,2,4]triazolo[1,5-*a*]pyrimidin-7-ol (**4o**)

Ethyl 3-oxobutanoate (1.0 g, 7.7 mmol) was taken in AcOH (5 mL) in a 50 mL round bottom flask under N_2_. To it was added 1*H*-1,2,4-triazol-5-amine (646 mg, 7.7 mmol). The reaction mixture was heated at 120 °C for 16 h. The reaction mixture was then evaporated to dryness using toluene as an azeotropic solvent and triturated with diethyl ether. This was finally dried under high vacuum which afforded **4o** as a white solid (610 mg, 53%). This was then used in the next step without any further purification. LCMS (ESI) *m*/*z* 151.06 [M+H^+^]; 78% (purity).

#### 4.5.27 5-Ethyl-[1,2,4]triazolo[1,5-*a*]pyrimidin-7-ol (**4p**)

Ethyl 3-oxopentanoate (200 mg, 1.4 mmol) was taken in AcOH (1.0 mL) in a 25 mL round bottom flask under N_2_. To it was added 1*H*-1,2,4-triazol-5-amine (139 mg, 1.6 mmol). The reaction mixture was heated at 120 °C for 16 h. The reaction mixture was then evaporated to dryness using toluene as an azeotropic solvent and triturated with diethyl ether. This was finally dried under high vacuum which afforded **4p** as an off-white solid (110 mg, 48%). This was then used in the next step without any further purification. LCMS(ESI) *m*/*z* 165.43 [M+H^+^]; 90% (purity).

#### 4.5.28 5-Cyclobutyl-[1,2,4]triazolo[1,5-*a*]pyrimidin-7-ol (**4r**)

Ethyl 3-cyclobutyl-3-oxopropanoate **2r** (5.0 g, 29.4 mmol) was taken in AcOH (25 mL) in a 100 mL round bottom flask under N_2_. To it was added 1*H*-1,2,4-triazol-5-amine (2.9 g, 35.2 mmol). The reaction mixture was heated at 110 °C for 14 h. The reaction mixture was then evaporated to dryness using toluene as an azeotropic solvent and triturated with diethyl ether. This was finally dried under high vacuum which afforded **4r** as a yellow semi-solid (3.8 g, crude). This was then used in the next step without any further purification. LCMS(ESI) *m*/*z* 191.30 [M+H^+^]; 84.60% (purity).

#### 4.5.29 5-Cyclopentyl-[1,2,4]triazolo[1,5-*a*]pyrimidin-7-ol (**4s**)

Ethyl 3-cyclopentyl-3-oxopropanoate (2.0 g, 10.8 mmol) was taken in AcOH (10 mL) in a 50 mL round bottom flask under N_2_. To it was added 1*H*-1,2,4-triazol-5-amine (1.08 g, 13.0 mmol). The reaction mixture was heated at 110 °C for 12 h. The reaction mixture was then evaporated to dryness using toluene as an azeotropic solvent and triturated with diethyl ether. This was finally dried under high vacuum which afforded **4s** as a brown solid (1.1 g, 50%). This was then used in the next step without any further purification. LCMS(ESI) *m*/*z* 205.05 [M+H^+^]; 66.67% (purity).

#### 4.5.30 5-Cyclohexyl-[1,2,4]triazolo[1,5-*a*]pyrimidin-7-ol (**4t**)

Ethyl 3-cyclohexyl-3-oxopropanoate (800 mg, 4.04 mmol) was taken in AcOH (4 mL) in a 50 mL round bottom flask under N_2_. To it was added 1*H*-1,2,4-triazol-5-amine (407 mg, 4.84 mmol). The reaction mixture was heated at 115 °C for 16 h. The reaction mixture was then evaporated to dryness using toluene as an azeotropic solvent and triturated with diethyl ether. This was finally dried under high vacuum which afforded **4t** as an off-white solid (410 mg, 46%). This was then used in the next step without any further purification. LCMS(ESI) *m*/*z* 217.30 [M−H^+^]; 52.65% (purity).

#### 4.5.31 7-Chloro-5-phenyl-[1,2,4]triazolo[1,5-*a*]pyrimidine (**5a**)

To a solution of 5-phenyl-[1,2,4]triazolo[1,5-*a*]pyrimidin-7-ol **4a** (10.0 g, 47.2 mmol) was added POCl_3_ (44 mL, 472.0 mmol) at 0 °C. The reaction mixture was then heated at 120 °C and monitored by TLC analysis (Hexane/EtOAc = 1:1). The reaction mixture was concentrated using toluene as an azeotropic solvent and quenched with ice cooled saturated NaHCO_3_ solution (20 mL) to pH 8. It was extracted with EtOAc (3 × 50 mL). The combined organic layer was dried over anhydrous Na_2_SO_4_ and concentrated under reduced pressure. The crude was further purified by flash chromatography on silica gel (60–120 mesh) using 15% hexane-EtOAc as eluent to afford **5a** as a pale yellow solid (4.2 g, 39%). ^1^H NMR (400 MHz, DMSO-*d*_6_): *δ* 8.77 (s, 1H), 8.38 (s, 1H), 8.32–8.34 (m, 2H), 7.60–7.61 (m, 3H). LCMS(ESI) *m*/*z* 231.03 [M+H^+^]; 92.15% (purity).

#### 4.5.32 7-Chloro-5-(4-methoxyphenyl)-[1,2,4]triazolo[1,5-*a*]pyrimidine (**5b**)

To a solution of 5-(4-methoxyphenyl)-[1,2,4]triazolo[1,5-*a*]pyrimidin-7-ol **4b** (500 mg, 2.1 mmol) was added POCl_3_ (1.6 mL, 16.8 mmol) at 0 °C. The reaction mixture was then heated at 100 °C and monitored by TLC analysis (Hexane/EtOAc = 1:1). The reaction mixture was concentrated using toluene as an azeotropic solvent and quenched with ice cooled saturated NaHCO_3_ solution (20 mL) to pH 8. It was extracted with EtOAc (3 × 30 mL). The combined organic layer was dried over anhydrous Na_2_SO_4_ and concentrated under reduced pressure. The crude was further purified by flash chromatography on silica gel (60–120 mesh) using 5% hexane-EtOAc as eluent to afford **5b** as a brown solid (210 mg, 39%). LCMS(ESI) *m*/*z* 261.15 [M+H^+^]; 85.81% (purity).

#### 4.5.33 7-Chloro-5-(2-methoxyphenyl)-[1,2,4]triazolo[1,5-*a*]pyrimidine (**5c**)

To a solution of 5-(2-methoxyphenyl)-[1,2,4]triazolo[1,5-*a*]pyrimidin-7-ol **4c** (500 mg, 2.1 mmol) was added POCl_3_ (5 mL, 52.5 mmol) at 0 °C. The reaction mixture was then heated at 100 °C and monitored by TLC analysis (Hexane/EtOAc = 1:1). Upon completion, the reaction mixture was concentrated using toluene as an azeotropic solvent and quenched with ice cooled saturated NaHCO_3_ solution (20 mL) to pH 8. It was extracted with EtOAc (3 × 100 mL). The combined organic layer was dried over anhydrous Na_2_SO_4_ and concentrated under reduced pressure to afford **5c**. This was then used in the next step without any further purification. LCMS(ESI) *m*/*z* 261.0 [M+H^+^]; 53.5% (purity).

#### 4.5.34 7-Chloro-5-(*p*-tolyl)-[1,2,4]triazolo[1,5-*a*]pyrimidine (**5d**)

To a solution of 5-(*p*-tolyl)-[1,2,4]triazolo[1,5-*a*]pyrimidin-7-ol **4d** (1.5 g, 6.6 mmol) was added POCl_3_ (20 mL, 211 mmol) at 0 °C. The reaction mixture was then heated at 80 °C and monitored by TLC analysis (Hexane/EtOAc = 1:1). Upon completion, the reaction mixture was concentrated using toluene as an azeotropic solvent and quenched with ice cooled saturated NaHCO_3_ solution (20 mL) to pH 8. It was extracted with EtOAc (3 × 100 mL). The combined organic layer was dried over anhydrous Na_2_SO_4_ and concentrated under reduced pressure. The crude was further purified by flash chromatography on silica gel (60–120 mesh) using 20% hexane-EtOAc as eluent to afford **5d** as a yellow solid (800 mg, 49%). LCMS(ESI) *m*/*z* 245.08 [M+H^+^]; 76.82% (purity).

#### 4.5.35 7-Chloro-5-(4-ethylphenyl)-[1,2,4]triazolo[1,5-*a*]pyrimidine (**5e**)

To a solution of 5-(4-ethylphenyl)-[1,2,4]triazolo[1,5-*a*]pyrimidin-7-ol **4e** (400 mg, 1.7 mmol) was added POCl_3_ (5 mL, 52.7 mmol) at 0 °C. The reaction mixture was then heated at 100 °C and monitored by TLC analysis (30%, EtOAc-hexane). Upon completion, the reaction mixture was concentrated using toluene as an azeotropic solvent and quenched with ice cooled saturated NaHCO_3_ solution (20 mL) to pH 8. It was extracted with EtOAc (3 × 25 mL). The combined organic layer was dried over anhydrous Na_2_SO_4_ and concentrated under reduced pressure. The crude was further purified by flash chromatography on silica gel (60–120 mesh) using 12% EtOAc-hexane as eluent to afford **5e** as a pale yellow solid (250 mg, 58%). LCMS(ESI) *m*/*z* 259.44 [M+H^+^]; 91.33% (purity).

#### 4.5.36 7-Chloro-5-(4-chlorophenyl)-[1,2,4]triazolo[1,5-*a*]pyrimidine (**5f**)

To a solution of 5-(4-chlorophenyl)-[1,2,4]triazolo[1,5-*a*]pyrimidin-7-ol **4f** (550 mg, 2.2 mmol) was added POCl_3_ (2.5 mL, 26.4 mmol) at 0 °C. The reaction mixture was then heated at 100 °C and monitored by TLC analysis (100% EtOAc). The reaction mixture was concentrated using toluene as an azeotropic solvent and quenched with ice cooled saturated NaHCO_3_ solution (20 mL) to pH 8. It was extracted with EtOAc (3 × 30 mL). The combined organic layer was dried over anhydrous Na_2_SO_4_ and concentrated under reduced pressure. The crude was triturated with diethyl ether to afford **5f** as an off-white solid (310 mg, 52%). ^1^H NMR (400 MHz, DMSO-*d*_6_): *δ* 8.78 (s, 1H), 8.42 (s, 1H), 8.35 (d, *J* = 8.4 Hz, 2H), 7.67 (d, *J* = 8.0 Hz, 2H).

#### 4.5.37 7-Chloro-5-(4-fluorophenyl)-[1,2,4]triazolo[1,5-*a*]pyrimidine (**5h**)

To a solution of 5-(4-fluorophenyl)-[1,2,4]triazolo[1,5-*a*]pyrimidin-7-ol **4h** (1.1 g, 4.8 mmol) was added POCl_3_ (6.0 mL, 62.4 mmol) at 0 °C. The reaction mixture was then heated at 100 °C and monitored by TLC analysis (100% EtOAc). The reaction mixture was concentrated using toluene as an azeotropic solvent and quenched with ice cooled saturated NaHCO_3_ solution (20 mL) to pH 8. It was extracted with EtOAc (3 × 30 mL). The combined organic layer was dried over anhydrous Na_2_SO_4_ and concentrated under reduced pressure. The crude was triturated with diethyl ether to afford **5h** as a yellow solid (635 mg, 53%). LCMS(ESI) *m*/*z* 249.07 [M+H^+^]; 75.08% (purity).

#### 4.5.38 7-Chloro-5-(2-fluorophenyl)-[1,2,4]triazolo[1,5-*a*]pyrimidine (**5i**)

To a solution of 5-(2-fluorophenyl)-[1,2,4]triazolo[1,5-*a*]pyrimidin-7-ol **4i** (800 mg, 3.5 mmol) was added POCl_3_ (10 mL, 112 mmol) at 0 °C. The reaction mixture was then heated at 100 °C and monitored by TLC analysis (30%, EtOAc-hexane). Upon completion, the reaction mixture was concentrated using toluene as an azeotropic solvent and quenched with ice cooled saturated NaHCO_3_ solution (30 mL) to pH 8. It was extracted with EtOAc (3 × 50 mL). The combined organic layer was dried over anhydrous Na_2_SO_4_ and concentrated under reduced pressure. The crude was further purified by flash chromatography on silica gel (60–120 mesh) using 14% EtOAc-hexane as eluent to afford **5i** as a pale yellow solid (100 mg, 11%). LCMS(ESI) *m*/*z* 248.90 [M+H^+^]; 50% (purity).

#### 4.5.39 7-Chloro-5-(4-(trifluoromethyl)phenyl)-[1,2,4]triazolo[1,5-*a*]pyrimidine (**5j**)

To a solution of 5-(4-(trifluoromethyl)phenyl)-[1,2,4]triazolo[1,5-*a*]pyrimidin-7-ol **4j** (3.0 g, 10.7 mmol) was added POCl_3_ (20 mL, 214 mmol) at 0 °C. The reaction mixture was then heated at 100 °C and monitored by TLC analysis (50%, EtOAc-hexane). Upon completion, the reaction mixture was concentrated using toluene as an azeotropic solvent and quenched with ice cooled saturated NaHCO_3_ solution (20 mL) to pH 8. It was extracted with EtOAc (3 × 25 mL). The combined organic layer was dried over anhydrous Na_2_SO_4_ and concentrated under reduced pressure. The crude was further purified by flash chromatography on silica gel (60–120 mesh) using 13% EtOAc-hexane as eluent to afford **5j** as a pale yellow solid (1.75 g, 55%). LCMS(ESI) *m*/*z* 299.08 [M+H^+^]; 93.67% (purity).

#### 4.5.40 7-Chloro-5-(pyridin-2-yl)-[1,2,4]triazolo[1,5-*a*]pyrimidine (**5k**)

To a solution of 5-(pyridin-2-yl)-[1,2,4]triazolo[1,5-*a*]pyrimidin-7-ol **4k** (4.0 g, 18.8 mmol) was added POCl_3_ (18 mL, 188 mmol) at 0 °C. The reaction mixture was then heated at 100 °C and monitored by TLC analysis (Hexane/EtOAc = 1:1). Upon completion, the reaction mixture was concentrated using toluene as an azeotropic solvent and quenched with ice cooled saturated NaHCO_3_ solution (20 mL) to pH 8. It was extracted with EtOAc (3 × 50 mL). The combined organic layer was dried over anhydrous Na_2_SO_4_ and concentrated under reduced pressure. The crude was further purified by flash chromatography on silica gel (60–120 mesh) using 20% hexane-EtOAc as eluent to afford **5k** as a yellow solid (1.5 g, 35%). LCMS(ESI) *m*/*z* 232.04 [M+H^+^]; 98.9% (purity).

#### 4.5.41 7-Chloro-5-(pyridin-3-yl)-[1,2,4]triazolo[1,5-*a*]pyrimidine (**5l**)

To a solution of 5-(pyridin-3-yl)-[1,2,4]triazolo[1,5-*a*]pyrimidin-7-ol **4l** (900 mg, 4.2 mmol) was added POCl_3_ (10 mL, 105 mmol) at 0 °C. The reaction mixture was then heated at 100 °C and monitored by TLC analysis (Hexane/EtOAc = 1:1). Upon completion, the reaction mixture was concentrated using toluene as an azeotropic solvent and quenched with ice cooled saturated NaHCO_3_ solution (20 mL) to pH 8. It was extracted with EtOAc (3 × 50 mL). The combined organic layer was dried over anhydrous Na_2_SO_4_ and concentrated under reduced pressure. The crude was further purified by flash chromatography on silica gel (60–120 mesh) using 20% hexane-EtOAc as eluent to afford **5l** as a yellow solid (480 mg, 49%). LCMS(ESI) *m*/*z* 232.19 [M+H^+^]; 74.46% (purity).

#### 4.5.42 7-Chloro-5-(pyridin-4-yl)-[1,2,4]triazolo[1,5-*a*]pyrimidine (**5m**)

To a solution of 5-(pyridin-4-yl)-[1,2,4]triazolo[1,5-*a*]pyrimidin-7-ol **4m** (500 mg, 2.3 mmol) was added POCl_3_ (3.0 mL, 34.0 mmol) at 0 °C. The reaction mixture was then heated at 85 °C and monitored by TLC analysis (100% EtOAc). The reaction mixture was concentrated using toluene as an azeotropic solvent and quenched with ice cooled saturated NaHCO_3_ solution (20 mL) to pH 8. It was extracted with EtOAc (3 × 50 mL). The combined organic layer was dried over anhydrous Na_2_SO_4_ and concentrated under reduced pressure. The crude was triturated with diethyl ether to afford **5m** as a yellow solid (260 mg, 48%). LCMS(ESI) *m*/*z* 232.18 [M+H^+^]; 72% (purity).

#### 4.5.43 5-Benzyl-7-chloro-[1,2,4]triazolo[1,5-*a*]pyrimidine (**5n**)

To a solution of 5-benzyl-[1,2,4]triazolo[1,5-*a*]pyrimidin-7-ol **4n** (900 mg, 3.9 mmol) was added POCl_3_ (4.5 mL, 47.8 mmol) at 0 °C. The reaction mixture was then heated at 100 °C and monitored by TLC analysis (100% EtOAc). The reaction mixture was concentrated using toluene as an azeotropic solvent and quenched with ice cooled saturated NaHCO_3_ solution (20 mL) to pH 8. It was extracted with EtOAc (3 × 30 mL). The combined organic layer was dried over anhydrous Na_2_SO_4_ and concentrated under reduced pressure. The crude was triturated with diethyl ether to afford **5n** as an off-white solid (610 mg, 62%). LCMS(ESI) *m*/*z* 245.22 [M+H^+^]; 94.26% (purity).

#### 4.5.44 7-Chloro-5-methyl-[1,2,4]triazolo[1,5-*a*]pyrimidine (**5o**)

To a solution of 5-methyl-[1,2,4]triazolo[1,5-*a*]pyrimidin-7-ol **4o** (600 mg, 4.0 mmol) was added POCl_3_ (2.0 mL, 22.0 mmol) at 0 °C. The reaction mixture was then heated at 100 °C and monitored by TLC analysis (50% EtOAc-hexane). The reaction mixture was concentrated using toluene as an azeotropic solvent and quenched with ice cooled saturated NaHCO_3_ solution (20 mL) to pH 8. It was extracted with EtOAc (3 × 30 mL). The combined organic layer was dried over anhydrous Na_2_SO_4_ and concentrated under reduced pressure. The crude was purified by flash chromatography on silica gel (100–200 mesh) using 20% EtOAc-hexane as eluent to afford **5o** as a white solid (340 mg, 50%). LCMS(ESI) *m*/*z* 169.03 [M+H^+^]; 92.15% (purity).

#### 4.5.45 7-Chloro-5-ethyl-[1,2,4]triazolo[1,5-*a*]pyrimidine (**5p**)

To a solution of 5-ethyl-[1,2,4]triazolo[1,5-*a*]pyrimidin-7-ol **4p** (100 mg, 0.6 mmol) was added POCl_3_ (1.0 mL, 10.8 mmol) at 0 °C. The reaction mixture was then heated at 100 °C and monitored by TLC analysis (50% EtOAc-hexane). The reaction mixture was concentrated using toluene as an azeotropic solvent and quenched with ice cooled saturated NaHCO_3_ solution (20 mL) to pH 8. It was extracted with EtOAc (3 × 15 mL). The combined organic layer was dried over anhydrous Na_2_SO_4_ and concentrated under reduced pressure. The crude was purified by flash chromatography on silica gel (100–200 mesh) using 12% EtOAc-hexane as eluent to afford **5p** as a white solid (85 mg, 76%).^1^H NMR (400 MHz, CDCl_3_): *δ* 8.51 (s, 1H), 7.11 (s, 1H), 2.98 (q, *J* = 7.6 Hz, 2H), 1.41 (t, *J* = 7.6 Hz, 3H).

#### 4.5.46 7-Chloro-5-cyclobutyl-[1,2,4]triazolo[1,5-*a*]pyrimidine (**5r**)

To a solution of 5-cyclobutyl-[1,2,4]triazolo[1,5-*a*]pyrimidin-7-ol **4r** (3.8 g, 20 mmol) was added POCl_3_ (30 mL, 320 mmol) at 0 °C. The reaction mixture was then heated at 80 °C and monitored by TLC analysis (Hexane/EtOAc = 1:1). Upon completion, the reaction mixture was concentrated using toluene as an azeotropic solvent and quenched with ice cooled saturated NaHCO_3_ solution (20 mL) to pH 8. It was extracted with EtOAc (3 × 100 mL). The combined organic layer was dried over anhydrous Na_2_SO_4_ and concentrated under reduced pressure. The crude was further purified by flash chromatography on silica gel (60–120 mesh) using 25% hexane-EtOAc as eluent to afford **5r** as a yellow semi-solid (1.8 g, 43%). LCMS(ESI) *m*/*z* 209.06 [M+H^+^]; 86.29% (purity).

#### 4.5.47 7-Chloro-5-cyclopentyl-[1,2,4]triazolo[1,5-*a*]pyrimidine (**5s**)

To a solution of 5-cyclopentyl-[1,2,4]triazolo[1,5-*a*]pyrimidin-7-ol **4s** (1.0 g, 4.9 mmol) was added POCl_3_ (6.0 mL, 63.7 mmol) at 0 °C. The reaction mixture was then heated at 100 °C and monitored by TLC analysis (100% EtOAc). The reaction mixture was concentrated using toluene as an azeotropic solvent and quenched with ice cooled saturated NaHCO_3_ solution (20 mL) to pH 8. It was extracted with EtOAc (3 × 50 mL). The combined organic layer was dried over anhydrous Na_2_SO_4_ and concentrated under reduced pressure. The crude was triturated with diethyl ether to afford **5s** as an off-white solid (270 mg, 25%). LCMS(ESI) *m*/*z* 223.09 [M+H^+^]; 69.45% (purity).

#### 4.5.48 7-Chloro-5-cyclohexyl-[1,2,4]triazolo[1,5-*a*]pyrimidine (**5t**)

To a solution of 5-cyclohexyl-[1,2,4]triazolo[1,5-*a*]pyrimidin-7-ol **4t** (400 mg, 1.8 mmol) was added POCl_3_ (2.0 mL, 21.6 mmol) at 0 °C. The reaction mixture was then heated at 100 °C and monitored by TLC analysis (100% EtOAc). The reaction mixture was concentrated using toluene as an azeotropic solvent and quenched with ice cooled saturated NaHCO_3_ solution (20 mL) to pH 8. It was extracted with EtOAc (3 × 50 mL). The combined organic layer was dried over anhydrous Na_2_SO_4_ and concentrated under reduced pressure. The crude was triturated with diethyl ether to afford **5t** as a yellow solid (110 mg, 25%). LCMS(ESI) *m*/*z* 237.0 [M+H^+^]; 61.42% (purity).

#### 4.5.49 *N*-(4-Methoxyphenethyl)-5-(4-methoxyphenyl)-[1,2,4]triazolo[1,5-*a*]pyrimidin-7-amine (**7**)

7-Chloro-5-(4-methoxyphenyl)-[1,2,4]triazolo[1,5-*a*]pyrimidine **5b** (200 mg, 0.77 mmol) was taken in NMP (2 mL) in a 50 mL round bottom flask under N_2_. To it was added 2-(4-methoxyphenyl)ethan-1-amine (127 mg, 0.84 mmol). The reaction mixture was heated at 100 °C and monitored by TLC analysis (EtOAc, 100%) until completion. The reaction mixture was then poured into ice water (30 g) and extracted with EtOAc (3 × 30 mL). The combined organic layer was dried over anhydrous Na_2_SO_4_ and concentrated under reduced pressure. The crude was purified by flash chromatography on silica gel (100–200 mesh) using 2% MeOH-DCM as eluent to afford **7** as a white solid (75 mg, 26%). M.P. 130–131 °C. ^1^H NMR (400 MHz, DMSO-*d*_6_): *δ* 8.52 (s, 1H), 8.37–8.40 (m, 1H), 8.15 (d, *J* = 8.8 Hz, 2H), 7.23 (d, *J* = 8.5 Hz, 2H), 7.07 (d, *J* = 8.9 Hz, 2H), 6.82–6.85 (m, 3H), 3.85 (s, 3H), 3.73–3.78 (m, 2H), 3.67 (s, 3H), 2.95 (m, 2H); ^13^C NMR (100 MHz, DMSO-*d*_6_): *δ* 161.3, 160.2, 157.8, 154.1, 152.8, 147.9, 130.6, 129.9, 129.3, 129.1, 113.9, 113.7, 84.9, 55.3, 54.9, 43.3, 33.7. LCMS (ESI) *m*/*z* 376.37.

#### 4.5.50 *N*-(4-Methoxyphenethyl)-5-(2-methoxyphenyl)-[1,2,4]triazolo[1,5-*a*]pyrimidin-7-amine (**8**)

7-Chloro-5-(2-methoxyphenyl)-[1,2,4]triazolo[1,5-*a*]pyrimidine **5c** (100 mg, 0.38 mmol) was taken in NMP (2 mL) in a 25 mL round bottom flask under N_2_. To it was added 2-(4-methoxyphenyl)ethan-1-amine (69 mg, 0.46 mmol) and the reaction mixture was stirred at rt for 30 min. The reaction mixture concentrated *in vacuo* and the resulting solid taken up in EtOAc and H_2_O, the organic layer was separated and the aqueous layer extracted with EtOAc (3 × 25 mL). The organic layers combined, washed with brine, dried over Na_2_SO_4_ and the solvent removed under reduced pressure. The crude was purified by flash chromatography on silica gel (100–200 mesh) using 30% EtOAc-hexane as eluent to afford **8** as an off-white solid (54 mg, 37%). M.P. 148–149 °C. ^1^H NMR (400 MHz, DMSO-*d*_6_): *δ* 8.45 (s, 1H), 8.33 (bs, 1H), 7.78 (d, *J* = 7.4 Hz, 1H), 7.45–7.49 (m, 1H), 7.20 (d, *J* = 8.2 Hz, 3H), 7.09 (t, *J* = 7.5 Hz, 1H), 6.84 (d, *J* = 8.9 Hz, 3H), 3.87 (s, 3H), 3.69 (s, 3H), 3.61–3.62 (m, 2H), 2.91–2.94 (m, 2H); ^13^C NMR (100 MHz, DMSO-*d*_6_): *δ* 159.6, 157.8, 157.0, 155.3, 154.5, 146.9, 131.1, 130.6, 130.5, 129.7, 127.5, 120.5, 113.8, 112.1, 89.3, 55.7, 54.9, 43.3, 33.4. LCMS (ESI) *m*/*z* 376.24.

#### 4.5.51 *N*-(4-Methoxyphenethyl)-5-(*p*-tolyl)-[1,2,4]triazolo[1,5-*a*]pyrimidin-7-amine (**9**)

7-Chloro-5-(*p*-tolyl)-[1,2,4]triazolo[1,5-*a*]pyrimidine **5d** (250 mg, 1.02 mmol) was taken in NMP (5 mL) in a 50 mL round bottom flask under N_2_. To it was added 2-(4-methoxyphenyl)ethan-1-amine (185 mg, 1.22 mmol) and the reaction mixture was stirred at rt for 20 min. The reaction mixture concentrated *in vacuo* and the resulting solid taken up in EtOAc and H_2_O, the organic layer was separated and the aqueous layer extracted with EtOAc (3 × 25 mL). The organic layers combined, washed with brine, dried over Na_2_SO_4_ and the solvent removed under reduced pressure. The crude was purified by flash chromatography on silica gel (100–200 mesh) using 30% EtOAc-hexane as eluent to afford **9** as an off-white solid (100 mg, 27%). M.P. 155–156 °C. ^1^H NMR (400 MHz, DMSO-*d*_6_): *δ* 8.44 (s, 1H), 8.28 (m, 1H), 8.07 (d, *J* = 7.8 Hz, 2H), 7.33 (d, *J* = 7.8 Hz, 2H), 7.23 (d, *J* = 8.1 Hz, 2H), 6.80–6.85 (m, 3H), 3.72–3.75 (m, 2H), 3.67 (s, 3H), 2.92–2.95 (m, 2H), 2.39 (s, 3H); ^13^C NMR (100 MHz, DMSO-*d*_6_): *δ* 160.2, 157.8, 155.5, 154.6, 147.8, 140.0, 134.8, 130.7, 129.9, 129.2, 127.3, 113.7, 84.4, 54.9, 43.2, 33.7, 20.9. LCMS (ESI) *m*/*z* 360.40.

#### 4.5.52 5-(4-*E*thylphenyl)-N-(4-methoxyphenethyl)-[1,2,4]triazolo[1,5-*a*]pyrimidin-7-amine (**10**)

7-Chloro-5-(4-ethylphenyl)-[1,2,4]triazolo[1,5-*a*]pyrimidine **5e** (100 mg, 0.38 mmol) was taken in NMP (3 mL) in a 50 mL round bottom flask under N_2_. To it was added 2-(4-methoxyphenyl)ethan-1-amine (70 mg, 0.46 mmol) and the reaction mixture was stirred at rt for 30 min. The reaction mixture concentrated *in vacuo* and the resulting solid taken up in EtOAc and H_2_O, the organic layer was separated and the aqueous layer extracted with EtOAc (3 × 25 mL). The organic layers combined, washed with brine, dried over Na_2_SO_4_ and the solvent removed under reduced pressure. The crude was purified by flash chromatography on silica gel (100–200 mesh) using 26% EtOAc-hexane as eluent to afford **10** as a pale yellow solid (78 mg, 54%). M.P. 151–12 °C. ^1^H NMR (400 MHz, DMSO-*d*_6_): *δ* 8.44 (s, 1H), 8.29 (t, *J* = 5.6 Hz, 1H), 8.08 (d, *J* = 8.2 Hz, 2H), 7.36 (d, *J* = 8.2 Hz, 2H), 7.23 (d, *J* = 8.5 Hz, 2H), 6.84 (d, *J* = 8.5 Hz, 2H), 6.79 (s, 1H), 3.72–3.77 (m, 2H), 3.66 (s, 3H), 2.91–2.95 (m, 2H), 2.68 (q, *J* = 7.6 Hz, 2H), 1.23 (t, *J* = 7.6 Hz, 3H); ^13^C NMR (100 MHz, DMSO-*d*_6_): *δ* 160.2, 157.8, 155.5, 154.6, 147.8, 146.2, 135.1, 130.7, 129.8, 127.9, 127.4, 113.7, 84.5, 54.9, 43.2, 33.7, 27.9, 15.4. LCMS (ESI) *m*/*z* 373.99.

#### 4.5.53 5-(4-Chlorophenyl)-N-(4-methoxyphenethyl)-[1,2,4]triazolo[1,5-*a*]pyrimidin-7-amine (**11**)

7-Chloro-5-(4-chlorophenyl)-[1,2,4]triazolo[1,5-*a*]pyrimidine **60** (200 mg, 0.77 mmol) was taken in NMP (2 mL) in a 50 mL round bottom flask under N_2_. To it was added 2-(4-methoxyphenyl)ethan-1-amine (127 mg, 0.84 mmol). The reaction mixture was heated at 100 °C and monitored by TLC analysis (EtOAc, 100%) until completion. The reaction mixture was then poured into ice water (30 g) and extracted with EtOAc (3 × 30 mL). The combined organic layer was dried over anhydrous Na_2_SO_4_ and concentrated under reduced pressure. The crude was purified by flash chromatography on silica gel (100–200 mesh) using 45% EtOAc-hexane as eluent to afford **11** as a white solid (110 mg, 30%). M.P. 178–180 °C. ^1^H NMR (400 MHz, DMSO-*d*_6_): *δ* 8.47 (s, 1H), 8.40 (bs, 1H), 8.20 (d, *J* = 8.5 Hz, 2H), 7.59 (d, *J* = 8.5 Hz, 2H), 7.23 (d, *J* = 8.4 Hz, 2H), 6.82–6.86 (m, 3H), 3.76–3.78 (m, 2H), 3.66 (s, 3H), 2.92–2.95 (m, 2H); ^13^C NMR (100 MHz, DMSO-*d*_6_): *δ* 158.8, 157.8, 155.4, 154.8, 147.9, 136.4, 135.1, 130.6, 129.9, 129.2, 128.6, 113.7, 84.8, 54.9, 43.3, 33.7. LCMS (ESI) *m*/*z* 380.34.

#### 4.5.54 5-(4-Fluorophenyl)-N-(4-methoxyphenethyl)-[1,2,4]triazolo[1,5-*a*]pyrimidin-7-amine (**13**)

7-Chloro-5-(4-fluorophenyl)-[1,2,4]triazolo[1,5-*a*]pyrimidine **5h** (125 mg, 0.5 mmol) was taken in NMP (3 mL) in a 50 mL round bottom flask under N_2_. To it was added 2-(4-methoxyphenyl)ethan-1-amine (91 mg, 0.6 mmol). The reaction mixture was stirred at rt and monitored by TLC analysis (70% EtOAc-hexane) until completion. The reaction mixture was then poured into ice water (30 g) and extracted with EtOAc (3 × 30 mL). The combined organic layer was dried over anhydrous Na_2_SO_4_ and concentrated under reduced pressure. The crude was purified by flash chromatography on silica gel (100–200 mesh) using 25% EtOAc-hexane as eluent to afford **13** as a white solid (40 mg, 22%). M.P. 153–155 °C. ^1^H NMR (400 MHz, DMSO-*d*_6_): *δ* 8.45 (s, 1H), 8.35–8.38 (m, 1H), 8.22–8.25 (m, 2H), 7.35 (t, *J* = 8.8 Hz, 2H), 7.23 (d, *J* = 8.4 Hz, 2H), 6.83 (d, *J* = 6.8 Hz, 3H), 3.73–3.78 (m, 2H), 3.66 (s, 3H), 2.92–2.95 (m, 2H); ^13^C NMR (100 MHz, DMSO-*d*_6_): *δ* 163.5 (d, *J* = 246 Hz), 159.8, 159.1, 157.8, 155.4, 154.7, 147.9, 134.1 (d, *J* = 3 Hz), 130.7, 129.8 (d, *J* = 8.5 Hz), 115.4 (d, *J* = 21 Hz), 113.7, 84.7, 54.9, 43.2, 33.8. LCMS (ESI) *m*/*z* 364.15.

#### 4.5.55 5-(2-Chlorophenyl)-N-(4-methoxyphenethyl)-[1,2,4]triazolo[1,5-*a*]pyrimidin-7-amine (**12**)

7-Chloro-5-(2-chlorophenyl)-[1,2,4]triazolo[1,5-*a*]pyrimidine (106 mg, 0.4 mmol) was taken in NMP (2 mL) in a 25 mL round bottom flask under N_2_. To it was added 2-(4-methoxyphenyl)ethan-1-amine (72 mg, 0.48 mmol) and the reaction mixture was stirred at rt for 30 min. The reaction mixture concentrated *in vacuo* and the resulting solid taken up in EtOAc and H_2_O, the organic layer was separated and the aqueous layer extracted with EtOAc (3 × 25 mL). The organic layers combined, washed with brine, dried over Na_2_SO_4_ and the solvent removed under reduced pressure. The crude was purified by flash chromatography on silica gel (100–200 mesh) using 27% EtOAc-hexane as eluent to afford **12** as a white solid (68 mg, 45%). ^1^H NMR (400 MHz, DMSO-*d*_6_): *δ* 8.52 (s, 2H), 7.60–7.51 (m, 2H), 7.50–7.46 (m, 2H), 7.18–7.16 (m, 2H), 6.82–6.80 (m, 2H), 6.55 (s, 1H), 3.68–3.64 (m, 5H), 2.91–2.88 (m, 2H); ^13^C NMR (100 MHz, DMSO-*d*_6_): *δ* 160.7, 157.8, 155.1, 154.7, 147.3, 138.4, 131.1, 131.1, 130.5, 130.5, 129.9, 129.8, 127.2, 113.8, 89.3, 54.9, 43.3, 33.7. LCMS (ESI) *m*/*z* 380.34.

#### 4.5.56 5-(2-Fluorophenyl)-N-(4-methoxyphenethyl)-[1,2,4]triazolo[1,5-*a*]pyrimidin-7-amine (**14**)

7-Chloro-5-(2-fluorophenyl)-[1,2,4]triazolo[1,5-*a*]pyrimidine **5i** (100 mg, 0.4 mmol) was taken in NMP (2 mL) in a 25 mL round bottom flask under N_2_. To it was added 2-(4-methoxyphenyl)ethan-1-amine (72 mg, 0.48 mmol) and the reaction mixture was stirred at rt for 30 min. The reaction mixture concentrated *in vacuo* and the resulting solid taken up in EtOAc and H_2_O, the organic layer was separated and the aqueous layer extracted with EtOAc (3 × 25 mL). The organic layers combined, washed with brine, dried over Na_2_SO_4_ and the solvent removed under reduced pressure. The crude was purified by flash chromatography on silica gel (100–200 mesh) using 27% EtOAc-hexane as eluent to afford **14** as a white solid (58 mg, 40%). M.P. 153–155 °C. ^1^H NMR (400 MHz, DMSO-*d*_6_): *δ* 8.50 (s, 2H), 7.93 (t, *J* = 6.7 Hz, 1H), 7.53–7.58 (m, 1H), 7.34–7.40 (m, 2H), 7.19 (d, *J* = 8.3 Hz, 2H), 6.82 (d, *J* = 8.4 Hz, 2H), 6.68 (s, 1H), 3.65–3.67 (m, 5H), 2.90–2.93 (m, 2H); ^13^C NMR (100 MHz, DMSO-*d*_6_): *δ* 159.8 (d, *J* = 248 Hz), 157.8, 156.9, 155.3, 154.8, 147.5, 131.8 (d, *J* = 8.7 Hz), 131.0, 130.4, 129.8, 126.4 (d, *J* = 11.2 Hz), 124.7 (d, *J* = 3.0 Hz), 116.4 (d, *J* = 22 Hz), 113.8, 88.5 (d, *J* = 8.0 Hz), 54.9, 43.4, 33.5. LCMS (ESI) *m*/*z* 364.33.

#### 4.5.57 *N*-(4-Methoxyphenethyl)-5-(4-(trifluoromethyl)phenyl)-[1,2,4]triazolo[1,5-*a*]pyrimidin-7-amine (**15**)

7-Chloro-5-(4-(trifluoromethyl)phenyl)-[1,2,4]triazolo[1,5-*a*]pyrimidine **5j** (100 mg, 0.33 mmol) was taken in NMP (2 mL) in a 25 mL round bottom flask under N_2_. To it was added 2-(4-methoxyphenyl)ethan-1-amine (62 mg, 0.4 mmol) and the reaction mixture was stirred at rt for 30 min. The reaction mixture concentrated *in vacuo* and the resulting solid taken up in EtOAc and H_2_O, the organic layer was separated and the aqueous layer extracted with EtOAc (3 × 25 mL). The organic layers combined, washed with brine, dried over Na_2_SO_4_ and the solvent removed under reduced pressure. The crude was purified by flash chromatography on silica gel (100–200 mesh) using 27% EtOAc-hexane as eluent to afford **15** as a pale yellow solid (75 mg, 54%). M.P. 191–193 °C. ^1^H NMR (400 MHz, DMSO-*d*_6_): *δ* 8.48–8.50 (m, 2H), 8.37 (d, *J* = 8.0 Hz, 2H), 7.89 (d, *J* = 8.3 Hz, 2H), 7.23 (d, *J* = 8.4 Hz, 2H), 6.92 (s, 1H), 6.83 (d, *J* = 8.4 Hz, 2H), 3.75–3.80 (m, 2H), 3.65 (s, 3H), 2.92–2.96 (m, 2H). LCMS (ESI) *m*/*z* 414.39.

#### 4.5.58 *N*-(4-Methoxyphenethyl)-5-(pyridin-2-yl)-[1,2,4]triazolo[1,5-*a*]pyrimidin-7-amine (**16**)

7-Chloro-5-(pyridin-2-yl)-[1,2,4]triazolo[1,5-*a*]pyrimidine **5k** (200 mg, 0.86 mmol) was taken in NMP (3 mL) in a 50 mL round bottom flask under N_2_. To it was added 2-(4-methoxyphenyl)ethan-1-amine (156 mg, 1.0 mmol) and the reaction mixture was stirred at 100 °C for 2 h. The reaction mixture concentrated *in vacuo* and the resulting solid taken up in EtOAc and H_2_O, the organic layer was separated and the aqueous layer extracted with EtOAc (3 × 20 mL). The organic layers combined, washed with brine, dried over Na_2_SO_4_ and the solvent removed under reduced pressure. The crude was purified by flash chromatography on silica gel (100–200 mesh) using 45% EtOAc-hexane as eluent to afford **16** as a white solid (80 mg, 27%). M.P. 172–173 °C. ^1^H NMR (400 MHz, DMSO-*d*_6_): *δ* 8.75 (d, *J* = 4.0 Hz, 1H), 8.50–8.51 (m, 2H), 8.45 (d, *J* = 8.0 Hz, 1H), 8.01 (t, *J* = 7.8 Hz, 1H), 7.53–7.56 (m, 1H), 7.34 (s, 1H), 7.21 (d, *J* = 8.5 Hz, 2H), 6.84 (d, *J* = 8.5 Hz, 2H), 3.69 (m, 2H), 3.67 (s, 3H), 2.93–2.97 (m, 2H); ^13^C NMR (100 MHz, DMSO-*d*_6_): *δ* 159.0, 157.8, 155.5, 154.9, 153.8, 149.2, 148.0, 137.4, 130.5, 129.7, 125.3, 121.4, 113.9, 84.5, 54.9, 43.5, 33.4. LCMS (ESI) *m*/*z* 347.39.

#### 4.5.59 *N*-(4-Methoxyphenethyl)-5-(pyridin-3-yl)-[1,2,4]triazolo[1,5-*a*]pyrimidin-7-amine (**17**)

7-Chloro-5-(pyridin-3-yl)-[1,2,4]triazolo[1,5-*a*]pyrimidine **5l** (200 mg, 0.86 mmol) was taken in NMP (5 mL) in a 50 mL round bottom flask under N_2_. To it was added 2-(4-methoxyphenyl)ethan-1-amine (156 mg, 1.04 mmol) and the reaction mixture was stirred at rt for 30 min. The reaction mixture concentrated *in vacuo* and the resulting solid taken up in EtOAc and H_2_O, the organic layer was separated and the aqueous layer extracted with EtOAc (3 × 25 mL). The organic layers combined, washed with brine, dried over Na_2_SO_4_ and the solvent removed under reduced pressure. The crude was purified by flash chromatography on silica gel (100–200 mesh) using 25% EtOAc-hexane as eluent to afford **17** as an off-white solid (60 mg, 17%). M.P. 152–153 °C. ^1^H NMR (400 MHz, DMSO-*d*_6_): *δ* 9.33 (s, 1H), 8.69–8.70 (m, 1H), 8.47–8.52 (m, 3H), 7.55–7.58 (m, 1H), 7.23 (d, *J* = 8.2 Hz, 2H), 6.94 (s, 1H), 6.82 (d, *J* = 8.2 Hz, 2H), 3.77 (m, 2H), 3.65 (s, 3H), 2.92–2.96 (m, 2H); ^13^C NMR (100 MHz, DMSO-*d*_6_): *δ* 157.9, 157.8, 155.4, 154.8, 150.9, 148.5, 148.1, 134.8, 133.1, 130.6, 129.9, 123.6, 113.7, 85.1, 54.9, 43.2, 33.8. LCMS (ESI) *m*/*z* 347.37.

#### 4.5.60 *N*-(4-Methoxyphenethyl)-5-(pyridin-4-yl)-[1,2,4]triazolo[1,5-*a*]pyrimidin-7-amine (**18**)

7-Chloro-5-(pyridin-4-yl)-[1,2,4]triazolo[1,5-*a*]pyrimidine **5m** (180 mg, 0.77 mmol) was taken in NMP (2 mL) in a 50 mL round bottom flask under N_2_. To it was added 2-(4-methoxyphenyl)ethan-1-amine (141 mg, 0.92 mmol). The reaction mixture was stirred at rt and monitored by TLC analysis (100% EtOAc) until completion. The reaction mixture was then poured into ice water (30 g) and extracted with EtOAc (3 × 30 mL). The combined organic layer was dried over anhydrous Na_2_SO_4_ and concentrated under reduced pressure. The crude was purified by flash chromatography on silica gel (100–200 mesh) using 3% MeOH-DCM as eluent to afford **18** as a white solid (70 mg, 26%). M.P. 217–219 °C. ^1^H NMR (400 MHz, DMSO-*d*_6_): *δ* 8.75 (d, *J* = 6.0 Hz, 2H), 8.52–8.56 (m, 2H), 8.11 (d, *J* = 6.0 Hz, 2H), 7.23 (d, *J* = 8.5 Hz, 2H), 6.96 (s, 1H), 6.82 (d, *J* = 8.5 Hz, 2H), 3.75–3.80 (m, 2H), 3.64 (s, 3H), 2.92–2.96 (m, 2H); ^13^C NMR (100 MHz, DMSO-*d*_6_): *δ* 157.8, 157.6, 155.4, 155.0, 150.2, 148.2, 144.6, 130.6, 129.9, 121.4, 113.7, 85.5, 54.9, 43.3, 33.8. LCMS (ESI) *m*/*z* 347.44.

#### 4.5.61 5-Benzyl-*N*-(4-methoxyphenethyl)-[1,2,4]triazolo[1,5-*a*]pyrimidin-7-amine (**19**)

5-Benzyl-7-chloro-[1,2,4]triazolo[1,5-*a*]pyrimidine **5n** (250 mg, 1.0 mmol) was taken in NMP (2 mL) in a 50 mL round bottom flask under N_2_. To it was added 2-(4-methoxyphenyl)ethan-1-amine (166 mg, 1.1 mmol). The reaction mixture was heated at 100 °C and monitored by TLC analysis (EtOAc, 100%) until completion. The reaction mixture was then poured into ice water (30 g) and extracted with EtOAc (3 × 30 mL). The combined organic layer was dried over anhydrous Na_2_SO_4_ and concentrated under reduced pressure. The crude was purified by flash chromatography on silica gel (100–200 mesh) using 35% EtOAc-hexane as eluent to afford **19** as an off-white solid (80 mg, 21%). M.P. 119–121 °C. ^1^H NMR (400 MHz, DMSO-*d*_6_): *δ* 8.38 (s, 1H), 8.27–8.31 (m, 1H), 7.28–7.34 (m, 4H), 7.19–7.23 (m, 1H), 7.15 (d, *J* = 8.1 Hz, 2H), 6.83 (d, *J* = 8.1 Hz, 2H), 6.36 (s, 1H), 3.99 (s, 2H), 3.70 (s, 3H), 3.52–3.57 (m, 2H), 2.83–2.86 (m, 2H); ^13^C NMR (100 MHz, DMSO-*d*_6_): *δ* 165.9, 157.8, 155.3, 154.2, 147.4, 138.9, 130.4, 129.8, 129.0, 128.3, 126.3, 113.8, 87.7, 54.9, 44.0, 43.3, 33.5. LCMS (ESI) *m*/*z* 360.48.

#### 4.5.62 *N*-(4-Methoxyphenethyl)-5-methyl-[1,2,4]triazolo[1,5-*a*]pyrimidin-7-amine (**20**)

7-Chloro-5-methyl-[1,2,4]triazolo[1,5-*a*]pyrimidine **5o** (280 mg, 1.7 mmol) was taken in NMP (3 mL) in a 50 mL round bottom flask under N_2_. To it was added 2-(4-methoxyphenyl)ethan-1-amine (301 mg, 2.0 mmol). The reaction mixture was heated at 100 °C and monitored by TLC analysis (EtOAc, 100%) until completion. The reaction mixture was then poured into ice water (30 g) and extracted with EtOAc (3 × 25 mL). The combined organic layer was dried over anhydrous Na_2_SO_4_ and concentrated under reduced pressure. The crude was purified by flash chromatography on silica gel (100–200 mesh) using 45% EtOAc-hexane as eluent to afford **20** as a white solid (130 mg, 27%). M.P. 129–130 °C. ^1^H NMR (400 MHz, DMSO-*d*_6_): *δ* 8.35 (s, 1H), 8.16 (bs, 1H), 7.20 (d, *J* = 8.2 Hz, 2H), 6.85 (d, *J* = 7.9 Hz, 2H), 6.31 (s, 1H), 3.71 (s, 3H), 3.53–3.56 (m, 2H), 2.86–2.90 (m, 2H), 2.41 (s, 3H); ^13^C NMR (100 MHz, DMSO-*d*_6_): *δ* 163.7, 157.8, 155.2, 154.0, 147.1, 130.5, 129.8, 113.7, 87.7, 54.9, 43.2, 33.5, 24.7. LCMS (ESI) *m*/*z* 284.44.

#### 4.5.63 Synthesis of 5-ethyl-*N*-(4-methoxyphenethyl)-[1,2,4]triazolo[1,5-*a*]pyrimidin-7-amine (**21**)

7-Chloro-5-ethyl-[1,2,4]triazolo[1,5-*a*]pyrimidine **5p** (60 mg, 0.32 mmol) was taken in NMP (1 mL) in a 25 mL round bottom flask under N_2_. To it was added 2-(4-methoxyphenyl)ethan-1-amine (59 mg, 0.39 mmol). The reaction mixture was stirred at rt and monitored by TLC analysis (70% EtOAc-hexane) until completion. The reaction mixture was then poured into ice water (30 g) and extracted with EtOAc (3 × 25 mL). The combined organic layer was dried over anhydrous Na_2_SO_4_ and concentrated under reduced pressure. The crude was purified by flash chromatography on silica gel (100–200 mesh) using 45% EtOAc-hexane as eluent to afford **21** as a white solid (34 mg, 35%). M.P. 70–72 °C. ^1^H NMR (400 MHz, DMSO-*d*_6_): *δ* 8.36 (s, 1H), 8.16 (bs, 1H), 7.19 (d, *J* = 7.7 Hz, 2H), 6.83 (d, *J* = 7.7 Hz, 2H), 6.24 (s, 1H), 3.69 (s, 3H), 3.56–3.58 (m, 2H), 2.86–2.88 (m, 2H), 2.63–2.67 (m, 2H), 1.21 (t, *J* = 7.5 Hz, 3H); ^13^C NMR (100 MHz, DMSO-*d*_6_): *δ* 168.4, 157.8, 155.3, 154.1, 147.3, 130.6, 129.8, 113.7, 86.8, 54.9, 43.2, 33.6, 31.1, 13.1. LCMS (ESI) *m*/*z* 298.15.

#### 4.5.64 5-Cyclobutyl-*N*-(4-methoxyphenethyl)-[1,2,4]triazolo[1,5-*a*]pyrimidin-7-amine (**23**)

7-Chloro-5-cyclobutyl-[1,2,4]triazolo[1,5-*a*]pyrimidine **5r** (200 mg, 0.96 mmol) was taken in NMP (4 mL) in a 50 mL round bottom flask under N_2_. To it was added 2-(4-methoxyphenyl)ethan-1-amine (174 mg, 1.1 mmol) and the reaction mixture was stirred at rt for 2 h. The reaction mixture concentrated *in vacuo* and the resulting solid taken up in EtOAc and H_2_O, the organic layer was separated and the aqueous layer extracted with EtOAc (3 × 20 mL). The organic layers combined, washed with brine, dried over Na_2_SO_4_ and the solvent removed under reduced pressure. The crude was purified by flash chromatography on silica gel (100–200 mesh) using 50% EtOAc-hexane as eluent to afford **23** as an off-white solid (22 mg, 7%). M.P. 99–100 °C. ^1^H NMR (400 MHz, DMSO-*d*_6_): *δ* 8.37 (s, 1H), 8.16 (bs, 1H), 7.18 (d, *J* = 8.1 Hz, 2H), 6.83 (d, *J* = 8.1 Hz, 2H), 6.12 (s, 1H), 3.69 (s, 3H), 3.54–3.59 (m, 3H), 2.85–2.89 (m, 2H), 2.20–2.32 (m, 4H), 1.94–2.01 (m, 1H), 1.82–1.83 (m, 1H); ^13^C NMR (100 MHz, DMSO-*d*_6_): *δ* 169.2, 157.8, 155.4, 154.1, 147.3, 130.6, 129.8, 113.7, 85.7, 54.9, 43.2, 41.6, 33.7, 27.3, 17.5. LCMS (ESI) *m*/*z* 324.36.

#### 4.5.65 5-Cyclopentyl-*N*-(4-methoxyphenethyl)-[1,2,4]triazolo[1,5-*a*]pyrimidin-7-amine (**24**)

7-Chloro-5-cyclopentyl-[1,2,4]triazolo[1,5-*a*]pyrimidine **5s** (260 mg, 1.2 mmol) was taken in NMP (4 mL) in a 50 mL round bottom flask under N_2_. To it was added 2-(4-methoxyphenyl)ethan-1-amine **5a** (212 mg, 1.4 mmol). The reaction mixture was stirred at rt and monitored by TLC analysis (50% EtOAc-hexane) until completion. The reaction mixture was then poured into ice water (30 g) and extracted with EtOAc (3 × 30 mL). The combined organic layer was dried over anhydrous Na_2_SO_4_ and concentrated under reduced pressure. The crude was purified by flash chromatography on silica gel (100–200 mesh) using 28% EtOAc-hexane as eluent to afford **24** as a white solid (90 mg, 23%). M.P. 99–100 °C. ^1^H NMR (400 MHz, DMSO-*d*_6_): *δ* 8.36 (s, 1H), 8.13 (t, *J* = 6.0 Hz, 1H), 7.19 (d, *J* = 8.5 Hz, 2H), 6.82 (d, *J* = 8.5 Hz, 2H), 6.18 (s, 1H), 3.69 (s, 3H), 3.56–3.61 (m, 2H), 3.06–3.09 (m, 1H), 2.85–2.89 (m, 2H), 1.91–1.95 (m, 2H), 1.75–1.76 (m, 4H), 1.61–1.62 (m, 2H); ^13^C NMR (100 MHz, DMSO-*d*_6_): *δ* 170.7, 157.8, 155.4, 154.1, 147.2, 130.6, 129.8, 113.7, 86.6, 54.9, 47.4, 43.3, 33.7, 32.5, 25.5. LCMS (ESI) *m*/*z* 338.36.

#### 4.5.66 5-Cyclohexyl-*N*-(4-methoxyphenethyl)-[1,2,4]triazolo[1,5-*a*]pyrimidin-7-amine (**25**)

7-Chloro-5-cyclohexyl-[1,2,4]triazolo[1,5-*a*]pyrimidine **5t** (100 mg, 0.42 mmol) was taken in NMP (2 mL) in a 50 mL round bottom flask under N_2_. To it was added 2-(4-methoxyphenyl)ethan-1-amine (76 mg, 0.51 mmol). The reaction mixture was stirred at rt and monitored by TLC analysis (100% EtOAc) until completion. The reaction mixture was then poured into ice water (30 g) and extracted with EtOAc (3 × 30 mL). The combined organic layer was dried over anhydrous Na_2_SO_4_ and concentrated under reduced pressure. The crude was purified by flash chromatography on silica gel (100–200 mesh) using 35% EtOAc-hexane as eluent to afford **25** as a white solid (40 mg, 27%). M.P. 193–194 °C. ^1^H NMR (400 MHz, DMSO-*d*_6_): *δ* 8.36 (s, 1H), 8.13 (t, *J* = 6.0 Hz, 1H), 7.19 (d, *J* = 8.4 Hz, 2H), 6.83 (d, *J* = 8.5 Hz, 2H), 6.14 (s, 1H), 3.69 (s, 3H), 3.57–3.62 (m, 2H), 2.85–2.89 (m, 2H), 2.58–2.59 (m, 1H), 1.77–1.80 (m, 4H), 1.69–1.72 (m, 1H), 1.45–1.54 (m, 2H), 1.18–1.38 (m, 3H); ^13^C NMR (100 MHz, DMSO-*d*_6_): *δ* 171.2, 157.8, 155.3, 154.1, 147.4, 130.7, 129.8, 113.7, 86.0, 54.9, 46.1, 43.3, 33.8, 31.7, 25.8, 25.5. LCMS (ESI) *m*/*z* 352.48.

#### 4.5.67 Ethyl 3-oxo-3-(pyridin-2-yl)propanoate (**26**)

Sodium hydride (2.9 g, 124 mmmol) was taken in dry THF (50 mL) in a 100 mL round bottom flask under N_2_ and cooled it down to 0 °C. To it was added a solution of 1-(pyridin-2-yl)ethan-1-one (5.0 g, 41.3 mmol) in THF (5 mL). The reaction mixture was stirred at rt for 30 min followed by the addition of diethyl carbonate (20 mL, 165 mmol). The reaction mixture was then stirred at rt for 12 h. Ice-cooled water was added dropwise to quench the reaction. It was extracted with EtOAc (3 × 100 mL). The combined organic layer was dried over anhydrous Na_2_SO_4_ and concentrated under reduced pressure. The crude was further triturated with diethyl ether which afforded **26** as a pale yellow semi-solid (4.0 g, 50%). LCMS(ESI) *m*/*z* 194.06 [M+H^+^]; 40% (purity).

#### 4.5.68 5-Phenyl-[1,2,4]triazolo[1,5-*a*]pyrimidine (**27**)

3-(Dimethylamino)-1-phenylpropan-1-one (300 mg, 1.7 mmol) was taken in AcOH (1 mL) in a 25 mL round bottom flask under N_2_. To it was added 1*H*-1,2,4-triazol-5-amine **2** (171 mg, 2.0 mmol). The reaction mixture was heated at 110 °C for 10 h. The reaction mixture was then evaporated to dryness using toluene as an azeotropic solvent. The crude was purified by flash chromatography on silica gel (100–200 mesh) using 15% EtOAc-hexane as eluent to afford **27** as a white solid (35 mg, 10%). M.P. 179–181 °C. ^1^H NMR (400 MHz, DMSO-*d*_6_): *δ* 9.47 (d, *J* = 7.2 Hz, 1H), 8.68 (s, 1H), 8.29–8.32 (m, 2H), 7.99 (d, *J* = 7.2 Hz, 1H), 7.57–7.61 (m, 3H); ^13^C NMR (100 MHz, DMSO-*d*_6_): *δ* 160.9, 156.4, 154.7, 137.6, 135.8, 131.5, 129.1, 127.7, 107.9. LCMS (ESI) *m*/*z* 197.28.

#### 4.5.69 5-Phenyl-[1,2,4]triazolo[1,5-*a*]pyrimidin-7-amine (**28**)

7-Chloro-5-phenyl-[1,2,4]triazolo[1,5-*a*]pyrimidine **6** (500 mg, 2.2 mmol) was taken sealed tube under N_2_. To it was added methanolic NH_3_ (20 mL) and the reaction mixture was heated at 120 °C for 16 h. The reaction mixture concentrated *in vacuo* and the crude was purified by flash chromatography on silica gel (100–200 mesh) using 5% MeOH-DCM as eluent to afford **28** as a yellow solid (410 mg, 63%). M.P. 302–304 °C. ^1^H NMR (400 MHz, DMSO-*d*_6_): *δ* 8.45 (s, 1H), 8.17 (bs, 2H), 8.05–8.07 (m, 2H), 7.49–7.54 (m, 3H), 6.78 (s, 1H); ^13^C NMR (100 MHz, DMSO-*d*_6_): *δ* 159.7, 155.9, 155.0, 149.3, 137.5, 130.2, 128.8, 126.9, 87.0. LCMS (ESI) *m*/*z* 212.14.

#### 4.5.70 *N*-Methyl-5-phenyl-[1,2,4]triazolo[1,5-*a*]pyrimidin-7-amine (**29**)

7-Chloro-5-phenyl-[1,2,4]triazolo[1,5-*a*]pyrimidine **6** (250 mg, 1.1 mmol) in NMP (3 mL) was taken sealed tube under N_2_. To it was added MeNH_2_ (2.0 M in THF) (0.6 mL, 1.3 mmol) and the reaction mixture was heated at 100 °C for 6 h. The reaction mixture concentrated *in vacuo* and the resulting solid taken up in EtOAc and H_2_O, the organic layer was separated and the aqueous layer extracted with EtOAc (3 × 30 mL). The organic layers combined, washed with brine, dried over Na_2_SO_4_ and the solvent removed under reduced pressure. The crude was purified by flash chromatography on silica gel (100–200 mesh) using 35% EtOAc-hexane as eluent to afford **29** as a white solid (84 mg, 34%). M.P. 200–202 °C. ^1^H NMR (400 MHz, DMSO-*d*_6_): *δ* 8.46 (s, 1H), 8.36–8.37 (m, 1H), 8.23 (d, *J* = 4.4 Hz, 2H), 7.53–7.54 (m, 3H), 6.87 (s, 1H), 3.10 (d, *J* = 4.4 Hz, 3H); ^13^C NMR (100 MHz, DMSO-*d*_6_): *δ* 160.3, 155.5, 154.7, 148.6, 137.6, 130.3, 128.6, 127.4, 84.6, 28.6. LCMS (ESI) *m*/*z* 224.28.

#### 4.5.71 *N*-Ethyl-5-phenyl-[1,2,4]triazolo[1,5-*a*]pyrimidin-7-amine (**30**)

7-Chloro-5-phenyl-[1,2,4]triazolo[1,5-*a*]pyrimidine **6** (250 mg, 1.1 mmol) in NMP (5 mL) was taken sealed tube under N_2_. To it was added EtNH_2_ (59 mg, 1.3 mmol) and the reaction mixture was heated at 100 °C for 6 h. The reaction mixture concentrated *in vacuo* and the resulting solid taken up in EtOAc and H_2_O, the organic layer was separated and the aqueous layer extracted with EtOAc (3 × 30 mL). The organic layers combined, washed with brine, dried over Na_2_SO_4_ and the solvent removed under reduced pressure. The crude was purified by flash chromatography on silica gel (100–200 mesh) using 35% EtOAc-hexane as eluent to afford **30** as a white solid (87 mg, 34%). M.P. 170–172 °C. ^1^H NMR (400 MHz, DMSO-*d*_6_): *δ* 8.46 (s, 1H), 8.38 (bs, 1H), 8.22–8.23 (m, 2H), 7.53 (m, 3H), 6.94 (s, 1H), 3.56–3.60 (m, 2H), 1.27 (t, *J* = 6.9 Hz, 3H); ^13^C NMR (100 MHz, DMSO-*d*_6_): *δ* 160.3, 155.6, 154.7, 147.8, 137.6, 130.2, 128.6, 127.4, 84.4, 36.5, 14.1. LCMS (ESI) *m*/*z* 240.40.

#### 4.5.72 5-Phenyl-*N*-propyl-[1,2,4]triazolo[1,5-*a*]pyrimidin-7-amine (**31**)

7-Chloro-5-phenyl-[1,2,4]triazolo[1,5-*a*]pyrimidine **6** (250 mg, 1.1 mmol) in NMP (3 mL) was taken sealed tube under N_2_. To it was added 1-propylamine (77 mg, 1.3 mmol) and the reaction mixture was heated at 90 °C for 2 h. The reaction mixture concentrated *in vacuo* and the resulting solid taken up in EtOAc and H_2_O, the organic layer was separated and the aqueous layer extracted with EtOAc (3 × 30 mL). The organic layers combined, washed with brine, dried over Na_2_SO_4_ and the solvent removed under reduced pressure. The crude was purified by flash chromatography on silica gel (100–200 mesh) using 32% EtOAc-hexane as eluent to afford **31** as a white solid (100 mg, 36%). M.P. 139–140 °C. ^1^H NMR (400 MHz, DMSO-*d*_6_): *δ* 8.46 (s, 1H), 8.40 (bs, 1H), 8.21–8.22 (m, 2H), 7.52 (m, 3H), 6.94 (s, 1H), 3.48–3.53 (m, 2H), 1.67–1.72 (m, 2H), 0.95 (t, *J* = 7.3 Hz, 3H); ^13^C NMR (100 MHz, DMSO-*d*_6_): *δ* 160.3, 155.6, 154.6, 148.0, 137.7, 130.2, 128.6, 127.4, 84.5, 43.2, 21.8, 11.2. LCMS (ESI) *m*/*z* found 252.30.

#### 4.5.73 *N*-Butyl-5-phenyl-[1,2,4]triazolo[1,5-*a*]pyrimidin-7-amine (**32**)

7-Chloro-5-phenyl-[1,2,4]triazolo[1,5-*a*]pyrimidine **6** (120 mg, 0.5 mmol) in NMP (2 mL) was taken sealed tube under N_2_. To it was added *n*-butylamine (45 mg, 0.6 mmol) and the reaction mixture was stirred at rt for 1 h. The reaction mixture concentrated *in vacuo* and the resulting solid taken up in EtOAc and H_2_O, the organic layer was separated and the aqueous layer extracted with EtOAc (3 × 30 mL). The organic layers combined, washed with brine, dried over Na_2_SO_4_ and the solvent removed under reduced pressure. The crude was purified by flash chromatography on silica gel (100–200 mesh) using 27% EtOAc-hexane as eluent to afford **32** as a white solid (40 mg, 28%). M.P. 125–126 °C. ^1^H NMR (400 MHz, DMSO-*d*_6_): *δ* 8.46 (s, 1H), 8.38 (t, *J* = 6.0 Hz, 1H), 8.21–8.23 (m, 2H), 7.52–7.54 (m, 3H), 6.93 (s, 1H), 3.51–3.56 (m, 2H), 1.62–1.70 (m, 2H), 1.35–1.44 (m, 2H), 0.93 (t, *J* = 7.3 Hz, 3H); ^13^C NMR (100 MHz, DMSO-*d*_6_): *δ* 160.3, 155.6, 154.7, 147.9, 137.6, 130.2, 128.6, 127.4, 84.5, 41.3, 30.5, 19.4, 13.7. LCMS (ESI) *m*/*z* 268.33.

#### 4.5.74 *N*-(2-Methoxyethyl)-5-phenyl-[1,2,4]triazolo[1,5-*a*]pyrimidin-7-amine (**33**)

7-Chloro-5-phenyl-[1,2,4]triazolo[1,5-*a*]pyrimidine **6** (250 mg, 1.1 mmol) in NMP (3 mL) was taken sealed tube under N_2_. To it was added 2-methoxyethan-1-amine (99 mg, 1.3 mmol) and the reaction mixture was heated at 90 °C for 2 h. The reaction mixture concentrated *in vacuo* and the resulting solid taken up in EtOAc and H_2_O, the organic layer was separated and the aqueous layer extracted with EtOAc (3 × 30 mL). The organic layers combined, washed with brine, dried over Na_2_SO_4_ and the solvent removed under reduced pressure. The crude was purified by flash chromatography on silica gel (100–200 mesh) using 45% EtOAc-hexane as eluent to afford **33** as a white solid (95 mg, 32%). M.P. 116–118 °C. ^1^H NMR (400 MHz, DMSO-*d*_6_): *δ* 8.47 (s, 1H), 8.27–8.28 (m, 1H), 8.21–8.23 (m, 2H), 7.53–7.54 (m, 3H), 7.02 (s, 1H), 3.71–3.74 (m, 2H), 3.61–3.63 (m, 2H), 3.29 (s, 3H); ^13^C NMR (100 MHz, DMSO-*d*_6_): *δ* 160.3, 155.5, 154.8, 148.2, 137.6, 130.3, 128.6, 127.4, 84.9, 70.2, 58.1, 41.4. LCMS (ESI) *m*/*z* 268.27.

#### 4.5.75 *N*-Cyclopropyl-5-phenyl-[1,2,4]triazolo[1,5-*a*]pyrimidin-7-amine (**34**)

7-Chloro-5-phenyl-[1,2,4]triazolo[1,5-*a*]pyrimidine **6** (120 mg, 0.5 mmol) in NMP (2 mL) was taken sealed tube under N_2_. To it was added cyclopropanamine (35 mg, 0.6 mmol) and the reaction mixture was stirred at rt for 1 h. The reaction mixture concentrated *in vacuo* and the resulting solid taken up in EtOAc and H_2_O, the organic layer was separated and the aqueous layer extracted with EtOAc (3 × 30 mL). The organic layers combined, washed with brine, dried over Na_2_SO_4_ and the solvent removed under reduced pressure. The crude was purified by flash chromatography on silica gel (100–200 mesh) using 25% EtOAc-hexane as eluent to afford **34** as a white solid (95 mg, 72%). M.P. 193–195 °C. ^1^H NMR (400 MHz, DMSO-*d*_6_): *δ* 8.77 (s, 1H), 8.47 (s, 1H), 8.17–8.20 (m, 2H), 7.54–7.58 (m, 3H), 7.03 (s, 1H), 2.84–2.89 (m, 1H), 0.88–0.95 (m, 2H), 0.76–0.80 (m, 2H); ^13^C NMR (100 MHz, DMSO-*d*_6_): *δ* 160.2, 155.6, 154.8, 147.3, 137.6, 130.4, 128.7, 127.3, 85.8, 23.8, 6.6. LCMS (ESI) *m*/*z* 252.19.

#### 4.5.76 *N*-Cyclobutyl-5-phenyl-[1,2,4]triazolo[1,5-*a*]pyrimidin-7-amine (**35**)

7-Chloro-5-phenyl-[1,2,4]triazolo[1,5-*a*]pyrimidine **6** (150 mg, 0.65 mmol) in NMP (5 mL) was taken sealed tube under N_2_. To it was added cyclobutanamine (55.4 mg, 0.78 mmol) and the reaction mixture was stirred at rt for 2 h. The reaction mixture concentrated *in vacuo* and the resulting solid taken up in EtOAc and H_2_O, the organic layer was separated and the aqueous layer extracted with EtOAc (3 × 30 mL). The organic layers combined, washed with brine, dried over Na_2_SO_4_ and the solvent removed under reduced pressure. The crude was purified by flash chromatography on silica gel (100–200 mesh) using 25% EtOAc-hexane as eluent to afford **35** as an off-white solid (50 mg, 29%). M.P. 193–194 °C. ^1^H NMR (400 MHz, DMSO-*d*_6_): *δ* 8.62 (d, *J* = 7.2 Hz, 1H), 8.48 (s, 1H), 8.21–8.23 (m, 2H), 7.53–7.54 (m, 3H), 6.87 (s, 1H), 4.43–4.53 (m, 1H), 2.38–2.44 (m, 2H), 2.23–2.32 (m, 2H), 1.71–1.78 (m, 2H); ^13^C NMR (100 MHz, DMSO-*d*_6_): *δ* 160.3, 155.7, 154.6, 146.8, 137.5, 130.3, 128.6, 127.4, 85.0, 46.6, 29.4, 14.6. LCMS (ESI) *m*/*z* 266.24.

#### 4.5.77 *N*-(2-Cyclohexylethyl)-5-phenyl-[1,2,4]triazolo[1,5-*a*]pyrimidin-7-amine (**36**)

7-Chloro-5-phenyl-[1,2,4]triazolo[1,5-*a*]pyrimidine **6** (250 mg, 1.1 mmol) in NMP (3 mL) was taken sealed tube under N_2_. To it was added 2-cyclohexylethan-1-amine (99 mg, 1.3 mmol) and the reaction mixture was heated at 90 °C for 2 h. The reaction mixture concentrated *in vacuo* and the resulting solid taken up in EtOAc and H_2_O, the organic layer was separated and the aqueous layer extracted with EtOAc (3 × 30 mL). The organic layers combined, washed with brine, dried over Na_2_SO_4_ and the solvent removed under reduced pressure. The crude was purified by flash chromatography on silica gel (100–200 mesh) using 45% EtOAc-hexane as eluent to afford **36** as an off-white solid (60 mg, 29%). M.P. 168–169 °C. ^1^H NMR (400 MHz, DMSO-*d*_6_): *δ* 8.46 (s, 1H), 8.32–8.35 (m, 1H), 8.20–8.22 (m, 2H), 7.53–7.54 (m, 3H), 6.89 (s, 1H), 3.52–3.57 (m, 2H), 1.77–1.80 (m, 2H), 1.55–1.68 (m, 5H), 1.38 (m, 1H), 1.12–1.13 (m, 3H), 0.90–0.98 (m, 2H); ^13^C NMR (100 MHz, DMSO-*d*_6_): *δ* 160.3, 154.8, 147.9, 137.7, 130.2, 128.6, 127.4, 84.5, 40.1, 35.6, 34.6, 32.6, 26.0, 25.7. LCMS (ESI) *m*/*z* 322.28.

#### 4.5.78 *N*-(4-Methoxyphenyl)-5-phenyl-[1,2,4]triazolo[1,5-*a*]pyrimidin-7-amine (**37**)

7-Chloro-5-phenyl-[1,2,4]triazolo[1,5-*a*]pyrimidine **6** (250 mg, 1.1 mmol) in NMP (5 mL) was taken sealed tube under N_2_. To it was added 4-methoxyaniline (140 mg, 1.2 mmol) and the reaction mixture was heated at 100 °C for 2 h. The reaction mixture concentrated *in vacuo* and the resulting solid taken up in EtOAc and H_2_O, the organic layer was separated and the aqueous layer extracted with EtOAc (3 × 30 mL). The organic layers combined, washed with brine, dried over Na_2_SO_4_ and the solvent removed under reduced pressure. The crude was purified by flash chromatography on silica gel (100–200 mesh) using 30% EtOAc-hexane as eluent to afford **37** as a white solid (135 mg, 39%). M.P. 200-202 °C. ^1^H NMR (400 MHz, DMSO-*d*_6_): *δ* 10.16 (s, 1H), 8.58 (s, 1H), 7.98 (bs, 2H), 7.44–7.50 (m, 5H), 7.08 (d, *J* = 8.0 Hz, 2H), 6.69 (s, 1H), 3.81 (s, 3H); ^13^C NMR (100 MHz, DMSO-*d*_6_): *δ* 160.5, 157.7, 155.9, 155.0, 147.3, 137.4, 130.3, 129.2, 128.8, 127.1, 126.6, 114.8, 85.6, 55.3. LCMS (ESI) *m*/*z* 318.38.

#### 4.5.79 *N*-(4-Methoxybenzyl)-5-phenyl-[1,2,4]triazolo[1,5-*a*]pyrimidin-7-amine (**38**)

7-Chloro-5-phenyl-[1,2,4]triazolo[1,5-*a*]pyrimidine **6** (250 mg, 1.1 mmol) in NMP (3 mL) was taken sealed tube under N_2_. To it was added (4-methoxyphenyl)methanamine (178 mg, 1.3 mmol) and the reaction mixture was heated at 100 °C for 1 h. The reaction mixture concentrated *in vacuo* and the resulting solid taken up in EtOAc and H_2_O, the organic layer was separated and the aqueous layer extracted with EtOAc (3 × 30 mL). The organic layers combined, washed with brine, dried over Na_2_SO_4_ and the solvent removed under reduced pressure. The crude was purified by flash chromatography on silica gel (100–200 mesh) using 35% EtOAc-hexane as eluent to afford **38** as a white solid (185 mg, 51%). M.P. 162-163 °C. ^1^H NMR (400 MHz, DMSO-*d*_6_): *δ* 8.98 (bs, 1H), 8.49 (s, 1H), 8.13–8.14 (m, 2H), 7.51 (m, 3H), 7.42 (d, *J* = 8.0 Hz, 2H), 6.89–6.92 (m, 3H), 4.71 (d, *J* = 6.2 Hz, 2H), 3.69 (s, 3H); ^13^C NMR (100 MHz, DMSO-*d*_6_): *δ* 160.1, 158.5, 155.6, 154.8, 147.9, 137.5, 130.3, 129.8, 128.7, 128.6, 127.3, 113.9, 85.1, 54.9, 44.0. LCMS (ESI) *m*/*z* 332.43.

#### 4.5.80 *N*-Phenethyl-5-phenyl-[1,2,4]triazolo[1,5-*a*]pyrimidin-7-amine (**39**)

7-Chloro-5-phenyl-[1,2,4]triazolo[1,5-*a*]pyrimidine **6** (250 mg, 1.1 mmol) was taken in NMP (5 mL) in a 50 mL round bottom flask under N_2_. To it was added 2-phenylethan-1-amine (140 mg, 1.2 mmol) and the reaction mixture was heated at 100 °C for 2 h. The reaction mixture concentrated *in vacuo* and the resulting solid taken up in EtOAc and H_2_O, the organic layer was separated and the aqueous layer extracted with EtOAc (3 × 30 mL). The organic layers combined, washed with brine, dried over Na_2_SO_4_ and the solvent removed under reduced pressure. The crude was purified by flash chromatography on silica gel (100–200 mesh) using 30% EtOAc-hexane as eluent to afford **39** as an off-white solid (210 mg, 61%). M.P. 164–165 °C. ^1^H NMR (400 MHz, DMSO-*d*_6_): *δ* 8.46 (s, 1H), 8.37–8.39 (m, 1H), 8.17–8.18 (m, 2H), 7.52–7.53 (m, 3H), 7.27–7.34 (m, 4H), 7.16–7.20 (m, 1H), 6.89 (s, 1H), 3.78–3.83 (m, 2H), 2.99–3.03 (m, 2H); ^13^C NMR (100 MHz, DMSO-*d*_6_): *δ* 160.7, 155.5, 154.7, 147.9, 138.9, 137.6, 130.2, 128.9, 128.6, 128.3, 127.4, 126.3, 84.8, 42.9, 34.6. LCMS (ESI) *m*/*z* 316.20.

#### 4.5.81 5-Phenyl-*N*-(3-phenylpropyl)-[1,2,4]triazolo[1,5-*a*]pyrimidin-7-amine (**40**)

7-Chloro-5-phenyl-[1,2,4]triazolo[1,5-*a*]pyrimidine **6** (120 mg, 0.5 mmol) was taken in NMP (3 mL) in a 50 mL round bottom flask under N_2_. To it was added 3-phenylpropan-1-amine (84 mg, 0.6 mmol) and the reaction mixture was stirred at rt for 30 min. The reaction mixture concentrated *in vacuo* and the resulting solid taken up in EtOAc and H_2_O, the organic layer was separated and the aqueous layer extracted with EtOAc (3 × 30 mL). The organic layers combined, washed with brine, dried over Na_2_SO_4_ and the solvent removed under reduced pressure. The crude was purified by flash chromatography on silica gel (100–200 mesh) using 30% EtOAc-hexane as eluent to afford **40** as a white solid (80 mg, 46%). M.P. 143–145 °C. ^1^H NMR (400 MHz, DMSO-*d*_6_): *δ* 8.46–8.49 (m, 2H), 8.15–8.17 (m, 2H), 7.52–7.57 (m, 3H), 7.25–7.30 (m, 4H), 7.17–7.21 (m, 1H), 6.81 (s, 1H), 3.53–3.58 (m, 2H), 2.69–2.73 (m, 2H), 1.95–2.03 (m, 2H). LCMS (ESI) *m*/*z* 330.20.

#### 4.5.82 5-Phenyl-*N*-(2-(pyridin-2-yl)ethyl)-[1,2,4]triazolo[1,5-*a*]pyrimidin-7-amine (**41**)

7-Chloro-5-phenyl-[1,2,4]triazolo[1,5-*a*]pyrimidine **6** (200 mg, 0.8 mmol) was taken in NMP (2 mL) in a 50 mL round bottom flask under N_2_. To it was added 2-(pyridin-2-yl)ethan-1-amine (127 mg, 1.0 mmol) and the reaction mixture was heated at 100 °C for 2 h. The reaction mixture concentrated *in vacuo* and the resulting solid taken up in EtOAc and H_2_O, the organic layer was separated and the aqueous layer extracted with EtOAc (3 × 30 mL). The organic layers combined, washed with brine, dried over Na_2_SO_4_ and the solvent removed under reduced pressure. The crude was purified by flash chromatography on silica gel (100–200 mesh) using 35% EtOAc-hexane as eluent to afford **41** as an off-white solid (135 mg, 49%). M.P. 111–114 °C. ^1^H NMR (400 MHz, DMSO-*d*_6_): *δ* 8.52 (d, *J* = 4.1 Hz, 1H), 8.46 (bs, 2H), 8.17–8.19 (m, 2H), 7.70 (t, *J* = 7.6 Hz, 1H), 7.53–7.56 (m, 3H), 7.21 (d, *J* = 7.8 Hz, 1H), 7.20–7.23 (m, 1H), 6.92 (s, 1H), 3.91–3.96 (m, 2H), 3.15–3.19 (m, 2H); ^13^C NMR (100 MHz, DMSO-*d*_6_): *δ* 160.2, 158.7, 155.5, 154.7, 149.1, 147.9, 137.6, 136.5, 130.3, 128.6, 127.4, 123.5, 121.7, 84.8, 41.3, 36.5. LCMS (ESI) *m*/*z* 317.19.

#### 4.5.83 5-Phenyl-*N*-(2-(pyridin-3-yl)ethyl)-[1,2,4]triazolo[1,5-*a*]pyrimidin-7-amine (**42**)

7-Chloro-5-phenyl-[1,2,4]triazolo[1,5-*a*]pyrimidine **6** (250 mg, 1.1 mmol) was taken in NMP (3 mL) in a 50 mL round bottom flask under N_2_. To it was added 2-(pyridin-3-yl)ethan-1-amine (159 mg, 1.3 mmol) and the reaction mixture was heated at 100 °C for 8 h. The reaction mixture concentrated *in vacuo* and the resulting solid taken up in EtOAc and H_2_O, the organic layer was separated and the aqueous layer extracted with EtOAc (3 × 30 mL). The organic layers combined, washed with brine, dried over Na_2_SO_4_ and the solvent removed under reduced pressure. The crude was purified by flash chromatography on silica gel (100–200 mesh) using 45% EtOAc-hexane as eluent to afford **42** as an off-white solid (146 mg, 42%). M.P. 169–171 °C. ^1^H NMR (400 MHz, DMSO-*d*_6_): *δ* 8.53 (d, *J* = 1.6 Hz, 1H), 8.43–8.47 (m, 2H), 8.38–8.40 (m, 1H), 8.17–8.20 (m, 2H), 7.74 (d, *J* = 7.9 Hz, 1H), 7.52–7.55 (m, 3H), 7.28–7.31 (m, 1H), 6.93 (s, 1H), 3.81–3.86 (m, 2H), 3.02–3.05 (m, 2H); ^13^C NMR (100 MHz, DMSO-*d*_6_): *δ* 160.3, 155.5, 154.7, 150.1, 147.9, 147.5, 137.6, 136.5, 134.3, 130.3, 128.6, 127.4, 123.3, 84.8, 42.4, 31.6. LCMS (ESI) *m*/*z* 317.48.

#### 4.5.84 5-Phenyl-*N*-(2-(pyridin-4-yl)ethyl)-[1,2,4]triazolo[1,5-*a*]pyrimidin-7-amine (**43**)

7-Chloro-5-phenyl-[1,2,4]triazolo[1,5-*a*]pyrimidine **6** (400 mg, 1.7 mmol) was taken in NMP (3 mL) in a 50 mL round bottom flask under N_2_. To it was added 2-(pyridin-4-yl)ethan-1-amine (256 mg, 2.1 mmol) and the reaction mixture was heated at 100 °C for 2 h. The reaction mixture concentrated *in vacuo* and the resulting solid taken up in EtOAc and H_2_O, the organic layer was separated and the aqueous layer extracted with EtOAc (3 × 30 mL). The organic layers combined, washed with brine, dried over Na_2_SO_4_ and the solvent removed under reduced pressure. The crude was purified by flash chromatography on silica gel (100–200 mesh) using 50% EtOAc-hexane as eluent to afford **43** as an off-white solid (120 mg, 22%). M.P. 231-233 °C. ^1^H NMR (400 MHz, DMSO-*d*_6_): *δ* 8.41–8.46 (m, 4H), 8.19–8.21 (m, 2H), 7.52–7.54 (m, 3H), 7.36 (d, *J* = 5.7 Hz, 2H), 6.96 (s, 1H), 3.83–3.88 (m, 2H), 3.03–3.06 (m, 2H); ^13^C NMR (100 MHz, DMSO-*d*_6_): *δ* 160.9, 160.3, 155.5, 154.7, 149.4, 147.9, 147.8, 137.6, 130.3, 128.6, 127.4, 124.4, 84.8, 41.7, 33.6. LCMS (ESI) *m*/*z* 317.38.

#### 4.5.85 *N*-(4-Methylphenethyl)-5-phenyl-[1,2,4]triazolo[1,5-*a*]pyrimidin-7-amine (**44**)

7-Chloro-5-phenyl-[1,2,4]triazolo[1,5-*a*]pyrimidine **6** (250 mg, 1.1 mmol) was taken in NMP (3 mL) in a 50 mL round bottom flask under N_2_. To it was added 2-(*p*-tolyl)ethan-1-amine (176 mg, 1.3 mmol) and the reaction mixture was heated at 100 °C for 2 h. The reaction mixture concentrated *in vacuo* and the resulting solid taken up in EtOAc and H_2_O, the organic layer was separated and the aqueous layer extracted with EtOAc (3 × 30 mL). The organic layers combined, washed with brine, dried over Na_2_SO_4_ and the solvent removed under reduced pressure. The crude was purified by flash chromatography on silica gel (100–200 mesh) using 30% EtOAc-hexane as eluent to afford **44** as a white solid (85 mg, 23%). M.P. 167–168 °C. ^1^H NMR (400 MHz, DMSO-*d*_6_): *δ* 8.46 (s, 1H), 8.35 (t, *J* = 6.0 Hz, 1H), 8.14–8.16 (m, 2H), 7.51–7.53 (m, 3H), 7.20 (d, *J* = 7.8 Hz, 2H), 7.08 (d, *J* = 7.8 Hz, 2H), 6.82 (s, 1H), 3.74–3.79 (m, 2H), 2.94–2.97 (m, 2H), 2.21 (s, 3H); ^13^C NMR (100 MHz, DMSO-*d*_6_): *δ* 160.2, 155.5, 154.7, 147.9, 137.6, 135.8, 135.2, 130.2, 128.9, 128.8, 128.5, 127.4, 84.8, 43.1, 34.3, 20.5. LCMS (ESI) *m*/*z* 330.39.

#### 4.5.86 *N*-(4-*E*thylphenethyl)-5-phenyl-[1,2,4]triazolo[1,5-*a*]pyrimidin-7-amine (**45**)

7-Chloro-5-phenyl-[1,2,4]triazolo[1,5-*a*]pyrimidine **6** (100 mg, 0.43 mmol) was taken in NMP (4 mL) in a 50 mL round bottom flask under N_2_. To it was added 2-(4-ethylphenyl)ethan-1-amine (78 mg, 0.52 mmol) and the reaction mixture was stirred at rt for 1 h. The reaction mixture concentrated *in vacuo* and the resulting solid taken up in EtOAc and H_2_O, the organic layer was separated and the aqueous layer extracted with EtOAc (3 × 30 mL). The organic layers combined, washed with brine, dried over Na_2_SO_4_ and the solvent removed under reduced pressure. The crude was purified by flash chromatography on silica gel (100–200 mesh) using 30% EtOAc-hexane as eluent to afford **45** as a pale yellow solid (107 mg, 72%). M.P. 152–154 °C. ^1^H NMR (400 MHz, DMSO-*d*_6_): *δ* 8.45 (s, 1H), 8.34 (bs, 1H), 8.15 (s, 2H), 7.31 (m, 3H), 7.21–7.23 (m, 2H), 7.09–7.11 (m, 2H), 6.80 (s, 1H), 3.77 (m, 2H), 2.96 (m, 2H), 2.49 (m, 2H), 1.08 (t, *J* = 7.5 Hz, 3H). LCMS (ESI) *m*/*z* 344.37.

#### 4.5.87 *N*-(4-Fluorophenethyl)-5-phenyl-[1,2,4]triazolo[1,5-*a*]pyrimidin-7-amine (**46**)

7-Chloro-5-phenyl-[1,2,4]triazolo[1,5-*a*]pyrimidine **6** (250 mg, 1.1 mmol) was taken in NMP (3 mL) in a 50 mL round bottom flask under N_2_. To it was added 2-(4-fluorophenyl)ethan-1-amine (181 mg, 1.3 mmol) and the reaction mixture was heated at 100 °C for 2 h. The reaction mixture concentrated *in vacuo* and the resulting solid taken up in EtOAc and H_2_O, the organic layer was separated and the aqueous layer extracted with EtOAc (3 × 30 mL). The organic layers combined, washed with brine, dried over Na_2_SO_4_ and the solvent removed under reduced pressure. The crude was purified by flash chromatography on silica gel (100–200 mesh) using 25% EtOAc-hexane as eluent to afford **46** as an off-white solid (120 mg, 33%). M.P. 169–170 °C. ^1^H NMR (400 MHz, DMSO-*d*_6_): *δ* 8.46 (s, 1H), 8.38 (t, *J* = 6.0 Hz, 1H), 8.16–8.19 (m, 2H), 7.52–7.53 (m, 3H), 7.34–7.38 (m, 2H), 7.01 (t, *J* = 8.8 Hz, 2H), 6.89 (s, 1H), 3.76–3.81 (m, 2H), 2.98–3.02 (m, 2H); ^13^C NMR (100 MHz, DMSO-*d*_6_): *δ* 160.9 (d, *J* = 240 Hz), 160.3, 155.5, 154.7, 147.9, 137.6, 135.0 (d, *J* = 3.0 Hz), 130.7 (d, *J* = 8.0 Hz), 130.2, 128.6, 127.4, 114.9 (d, *J* = 21 Hz), 84.8, 42.9, 33.7. LCMS (ESI) *m*/*z* 334.37.

#### 4.5.88 5-Phenyl-*N*-(4-(trifluoromethyl)phenethyl)-[1,2,4]triazolo[1,5-*a*]pyrimidin-7-amine (**47**)

7-Chloro-5-phenyl-[1,2,4]triazolo[1,5-*a*]pyrimidine **6** (150 mg, 0.65 mmol) was taken in NMP (3 mL) in a 50 mL round bottom flask under N_2_. To it was added 2-(4-(trifluoromethyl)phenyl)ethan-1-amine (147 mg, 0.78 mmol) and the reaction mixture was heated at 100 °C for 2 h. The reaction mixture concentrated *in vacuo* and the resulting solid taken up in EtOAc and H_2_O, the organic layer was separated and the aqueous layer extracted with EtOAc (3 × 30 mL). The organic layers combined, washed with brine, dried over Na_2_SO_4_ and the solvent removed under reduced pressure. The crude was purified by flash chromatography on silica gel (100–200 mesh) using 50% EtOAc-hexane as eluent to afford **47** as a light colour solid (120 mg, 20%). M.P. 183–184 °C. ^1^H NMR (400 MHz, DMSO-*d*_6_): *δ* 8.43–8.46 (m, 2H), 8.16–8.17 (m, 2H), 7.63–7.65 (m, 2H), 7.52–7.56 (m, 5H), 6.89 (s, 1H), 3.84–3.85 (m, 2H), 3.09–3.13 (m, 2H); ^13^C NMR (100 MHz, DMSO-*d*_6_): *δ* 160.3, 155.4, 154.7, 147.8, 143.9, 137.5, 130.2, 129.7, 128.5, 127.4, 127.2, 125.0 (q, *J* = 4.0 Hz), 124.3 (q, *J* = 270 Hz), 84.8, 42.5, 34.3. LCMS(ESI) *m*/*z* 384.38.

#### 4.5.89 5-Phenyl-*N*-(4-(trifluoromethoxy)phenethyl)-[1,2,4]triazolo[1,5-*a*]pyrimidin-7-amine (**48**)

7-Chloro-5-phenyl-[1,2,4]triazolo[1,5-*a*]pyrimidine **6** (150 mg, 0.65 mmol) was taken in NMP (5 mL) in a 50 mL round bottom flask under N_2_. To it was added 2-(4-(trifluoromethoxy)phenyl)ethan-1-amine (267 mg, 0.78 mmol) and the reaction mixture was heated at 100 °C for 2 h. The reaction mixture concentrated *in vacuo* and the resulting solid taken up in EtOAc and H_2_O, the organic layer was separated and the aqueous layer extracted with EtOAc (3 × 30 mL). The organic layers combined, washed with brine, dried over Na_2_SO_4_ and the solvent removed under reduced pressure. The crude was purified by flash chromatography on silica gel (100–200 mesh) using 25% EtOAc-hexane as eluent to afford **48** as an off-white solid (170 mg, 39%). M.P. 179–180 °C. ^1^H NMR (400 MHz, DMSO-*d*_6_): *δ* 8.46 (s, 1H), 8.39–8.42 (m, 1H), 8.17–8.18 (m, 2H), 7.52–7.53 (m, 3H), 7.45 (d, *J* = 8.3 Hz, 2H), 7.27 (d, *J* = 8.0 Hz, 2H), 6.90 (s, 1H), 3.79–3.84 (m, 2H), 3.03–3.06 (m, 2H); ^13^C NMR (100 MHz, DMSO-*d*_6_): *δ* 160.3, 155.5, 154.7, 147.9, 146.9, 138.4, 137.6, 130.7, 130.2, 128.5, 127.4, 120.8, 120.0 (q, *J* = 256 Hz), 84.8, 42.7, 33.8. LCMS (ESI) *m*/*z* 400.43.

#### 4.5.90 2-(4-Methoxyphenyl)-*N*-(5-phenyl-[1,2,4]triazolo[1,5-*a*]pyrimidin-7-yl)acetamide (**49**)

5-phenyl-[1,2,4]triazolo[1,5-*a*]pyrimidin-7-amine **28** (150 mg, 0.71 mmol) was taken in DMF (5 mL) in a 50 mL round bottom flask under N_2_ and cooled it to 0 °C. To it were sequentially added 2-(4-methoxyphenyl)acetic acid (141 mg, 0.85 mmol), EDCI.HCl (216 mg, 1.1 mmol), HOBt (148 mg, 1.1 mmol) and DIPEA (181 mg, 1.4 mmol). The reaction mixture was then heated at 90 °C for 24 h. The reaction mixture concentrated *in vacuo* and the resulting solid taken up in EtOAc and H_2_O, the organic layer was separated and the aqueous layer extracted with EtOAc (3 × 30 mL). The organic layers combined, washed with brine, dried over Na_2_SO_4_ and the solvent removed under reduced pressure. The crude was purified by flash chromatography on silica gel (100–200 mesh) using 35% EtOAc-hexane as eluent to afford **49** as an off-white solid (70 mg, 27%). M.P. 220–222 °C. ^1^H NMR (400 MHz, DMSO-*d*_6_): *δ* 11.59 (s, 1H), 8.72 (s, 1H), 8.41 (s, 1H), 8.11–8.12 (m, 2H), 7.57–7.58 (m, 3H), 7.31 (d, *J* = 8.4 Hz, 2H), 6.91 (d, *J* = 8.5 Hz, 2H), 4.02 (s, 2H), 3.73 (s, 3H); ^13^C NMR (100 MHz, DMSO-*d*_6_): *δ* 172.1, 161.5, 158.2, 155.2, 142.1, 136.7, 130.9, 130.5, 129.0, 127.3, 126.4, 113.8, 94.1, 55.0, 41.9. LCMS (ESI) *m*/*z* 360.35.

#### 4.5.91 2-(3,4-Dimethoxyphenyl)-*N*-(5-phenyl-[1,2,4]triazolo[1,5-*a*]pyrimidin-7-yl)acetamide (**50**)

5-Phenyl-[1,2,4]triazolo[1,5-*a*]pyrimidin-7-amine **28** (120 mg, 0.57 mmol) was taken in THF:CHCl_3_ (1:2, 6 mL) in a 50 mL round bottom flask under N_2_ and cooled it to 0 °C. To it were sequentially added 2-(3,4-dimethoxyphenyl)acetic acid (334 mg, 1.7 mmol), EDCI.HCl (163 mg, 0.85 mmol), HOBt (116 mg, 0.85 mmol) and DIPEA (181 mg, 1.4 mmol). The reaction mixture was then heated at 100 °C for 2 h. The reaction mixture concentrated *in vacuo* and the resulting solid taken up in EtOAc and H_2_O, the organic layer was separated and the aqueous layer extracted with EtOAc (3 × 30 mL). The organic layers combined, washed with brine, dried over Na_2_SO_4_ and the solvent removed under reduced pressure. The crude was purified by flash chromatography on silica gel (100–200 mesh) using 22% EtOAc-hexane as eluent to afford **50** as a white solid (38 mg, 17%). M.P. 202–204 °C. ^1^H NMR (400 MHz, DMSO-*d*_6_): *δ* 11.56 (s, 1H), 8.72 (s, 1H), 8.41 (s, 1H), 8.12 (m, 2H), 7.58 (m, 3H), 7.01 (s, 1H), 6.91 (m, 2H), 4.00 (s, 2H), 3.75 (s, 3H), 3.73 (s, 3H); ^13^C NMR (100 MHz, DMSO-*d*_6_): *δ* 171.9, 161.5, 155.3, 155.2, 148.6, 147.8, 142.1, 136.7, 131.1, 129.1, 127.3, 126.9, 121.6, 113.4, 111.9, 94.2, 55.5, 55.4, 42.4. LCMS (ESI) *m*/*z* 390.08.

#### 4.5.92 *N*-Phenethyl-5-(pyridin-2-yl)-[1,2,4]triazolo[1,5-*a*]pyrimidin-7-amine (**51**)

7-Chloro-5-(pyridin-2-yl)-[1,2,4]triazolo[1,5-*a*]pyrimidine **5k** (100 mg, 0.43 mmol) was taken in NMP (4 mL) in a 50 mL round bottom flask under N_2_. To it was added 2-phenylethan-1-amine (63 mg, 0.52 mmol) and the reaction mixture was stirred at rt for 30 min. The reaction mixture concentrated *in vacuo* and the resulting solid taken up in EtOAc and H_2_O, the organic layer was separated and the aqueous layer extracted with EtOAc (3 × 20 mL). The organic layers combined, washed with brine, dried over Na_2_SO_4_ and the solvent removed under reduced pressure. The crude was purified by flash chromatography on silica gel (100–200 mesh) using 50% EtOAc-hexane as eluent to afford **51** as an off-white solid (60 mg, 44%). M.P. 214–215 °C. ^1^H NMR (400 MHz, DMSO-*d*_6_): *δ* 8.75 (d, *J* = 4.0 Hz, 1H), 8.52 (bs, 2H), 8.46 (d, *J* = 7.8 Hz, 1H), 8.01 (t, *J* = 7.8 Hz, 1H), 7.53–7.56 (m, 1H), 7.37 (s, 1H), 7.27–7.30 (m, 4H), 7.19–7.20 (m, 1H), 3.73–3.75 (m, 2H), 3.01–3.05 (m, 2H); ^13^C NMR (100 MHz, DMSO-*d*_6_): *δ* 159.0, 155.5, 154.9, 153.8, 149.2, 148.0, 138.6, 137.4, 128.7, 128.4, 126.3, 125.2, 121.4, 84.4, 43.2, 34.2. LCMS (ESI) *m*/*z* 317.22.

#### 4.5.93 5-(Pyridin-2-yl)-*N*-(2-(pyridin-2-yl)ethyl)-[1,2,4]triazolo[1,5-*a*]pyrimidin-7-amine (**52**)

7-Chloro-5-(pyridin-2-yl)-[1,2,4]triazolo[1,5-*a*]pyrimidine **5k** (100 mg, 0.43 mmol) was taken in NMP (4 mL) in a 50 mL round bottom flask under N_2_. To it was added 2-(pyridin-2-yl)ethan-1-amine (63 mg, 0.52 mmol) and the reaction mixture was stirred at rt for 1 h. The reaction mixture concentrated *in vacuo* and the resulting solid taken up in EtOAc and H_2_O, the organic layer was separated and the aqueous layer extracted with EtOAc (3 × 20 mL). The organic layers combined, washed with brine, dried over Na_2_SO_4_ and the solvent removed under reduced pressure. The crude was purified by flash chromatography on silica gel (100–200 mesh) using 30% EtOAc-hexane as eluent to afford **52** as a pale yellow solid (52 mg, 38%). M.P. 156–157 °C. ^1^H NMR (400 MHz, DMSO-*d*_6_): *δ* 8.75–8.76 (m, 1H), 8.58 (bs, 1H), 8.51–8.53 (m, 2H), 8.45 (d, *J* = 7.9 Hz, 1H), 7.98–8.02 (m, 1H), 7.67–7.71 (m, 1H), 7.53–7.56 (m, 1H), 7.37 (s, 1H), 7.33 (d, *J* = 7.8 Hz, 1H), 7.19–7.22 (m, 1H), 3.89–3.91 (m, 2H), 3.16–3.20 (m, 2H); ^13^C NMR (100 MHz, DMSO-*d*_6_): *δ* 159.0, 158.4, 155.5, 154.9, 153.8, 149.2, 149.1, 148.0, 137.4, 136.6, 125.2, 123.4, 121.7, 121.3, 84.4, 41.5, 36.3. LCMS (ESI) *m*/*z* 318.27.

#### 4.5.94 5-(Pyridin-2-yl)-*N*-(2-(pyridin-3-yl)ethyl)-[1,2,4]triazolo[1,5-*a*]pyrimidin-7-amine (**53**)

7-Chloro-5-(pyridin-2-yl)-[1,2,4]triazolo[1,5-*a*]pyrimidine **5k** (100 mg, 0.43 mmol) was taken in NMP (4 mL) in a 50 mL round bottom flask under N_2_. To it was added 2-(pyridin-3-yl)ethan-1-amine **5i** (63 mg, 0.52 mmol) and the reaction mixture was stirred at rt for 1 h. The reaction mixture concentrated *in vacuo* and the resulting solid taken up in EtOAc and H_2_O, the organic layer was separated and the aqueous layer extracted with EtOAc (3 × 15 mL). The organic layers combined, washed with brine, dried over Na_2_SO_4_ and the solvent removed under reduced pressure. The crude was purified by flash chromatography on silica gel (100–200 mesh) using 30% EtOAc-hexane as eluent to afford **53** as a pale yellow solid (104 mg, 76%). M.P. 212–213 °C. ^1^H NMR (400 MHz, DMSO-*d*_6_): *δ* 8.74–8.75 (m, 1H), 8.56–8.59 (m, 1H), 8.50–8.52 (m, 2H), 8.46 (d, *J* = 7.9 Hz, 1H), 8.39–8.40 (m, 1H), 7.99–8.03 (m, 1H), 7.73 (d, *J* = 7.8 Hz, 1H), 7.54–7.56 (m, 1H), 7.37 (s, 1H), 7.29–7.32 (m, 1H), 3.77–3.82 (m, 2H), 3.03–3.07 (m, 2H); ^13^C NMR (100 MHz, DMSO-*d*_6_): *δ* 159.0, 155.5, 154.9, 153.8, 149.9, 149.2, 148.0, 147.6, 137.4, 136.3, 134.2, 125.2, 123.4, 121.4, 84.5, 42.7, 31.3. LCMS (ESI) *m*/*z* 318.37.

#### 4.5.95 5-(Pyridin-2-yl)-*N*-(2-(pyridin-4-yl)ethyl)-[1,2,4]triazolo[1,5-*a*]pyrimidin-7-amine (**54**)

7-Chloro-5-(pyridin-2-yl)-[1,2,4]triazolo[1,5-*a*]pyrimidine **5k** (100 mg, 0.43 mmol) was taken in NMP (4 mL) in a 50 mL round bottom flask under N_2_. To it was added 2-(pyridin-4-yl)ethan-1-amine (63 mg, 0.52 mmol) and the reaction mixture was stirred at rt for 1 h. The reaction mixture concentrated *in vacuo* and the resulting solid taken up in EtOAc and H_2_O, the organic layer was separated and the aqueous layer extracted with EtOAc (3 × 15 mL). The organic layers combined, washed with brine, dried over Na_2_SO_4_ and the solvent removed under reduced pressure. The crude was purified by flash chromatography on silica gel (100–200 mesh) using 30% EtOAc-hexane as eluent to afford **54** as a pale yellow solid (70 mg, 51%). M.P. 201–202 °C. ^1^H NMR (400 MHz, DMSO-*d*_6_): *δ* 8.75 (d, *J* = 4.3 Hz, 1H), 8.57 (bs, 1H), 8.51 (s, 1H), 8.45–8.46 (m, 3H), 7.99–8.03 (m, 1H), 7.54–7.57 (m, 1H), 7.38 (s, 1H), 7.34 (d, *J* = 7.6 Hz, 2H), 3.81–3.82 (m, 2H), 3.04–3.08 (m, 2H); ^13^C NMR (100 MHz, DMSO-*d*_6_): *δ* 159.1, 155.5, 155.0, 153.8, 149.5, 149.2, 148.0, 147.7, 137.4, 125.3, 124.3, 121.4, 84.5, 42.0, 33.3. LCMS (ESI) *m*/*z* 318.27.

#### 4.5.96 *N*-(4-Methylphenethyl)-5-(pyridin-2-yl)-[1,2,4]triazolo[1,5-*a*]pyrimidin-7-amine (**55**)

7-Chloro-5-(pyridin-2-yl)-[1,2,4]triazolo[1,5-*a*]pyrimidine **5k** (100 mg, 0.43 mmol) was taken in NMP (4 mL) in a 50 mL round bottom flask under N_2_. To it was added 2-(*p*-tolyl)ethan-1-amine (70 mg, 0.52 mmol) and the reaction mixture was stirred at rt for 10 min. The reaction mixture concentrated *in vacuo* and the resulting solid taken up in EtOAc and H_2_O, the organic layer was separated and the aqueous layer extracted with EtOAc (3 × 10 mL). The organic layers combined, washed with brine, dried over Na_2_SO_4_ and the solvent removed under reduced pressure. The crude was purified by flash chromatography on silica gel (100–200 mesh) using 30% EtOAc-hexane as eluent to afford **55** as a pale yellow solid (60 mg, 42%). M.P. 194–196 °C. ^1^H NMR (400 MHz, DMSO-*d*_6_): *δ* 8.75 (d, *J* = 4.0 Hz, 1H), 8.51 (bs, 2H), 8.45 (d, *J* = 7.9 Hz, 1H), 7.98–8.02 (m, 1H), 7.53–7.56 (m, 1H), 7.34 (s, 1H), 7.18 (d, *J* = 7.8 Hz, 2H), 7.08 (d, *J* = 7.7 Hz, 2H), 3.71 (m, 2H), 2.96–2.99 (m, 2H), 2.21 (s, 3H); ^13^C NMR (100 MHz, DMSO-*d*_6_): *δ* 159.0, 155.5, 154.9, 153.8, 149.2, 148.0, 137.4, 135.5, 135.3, 128.9, 128.6, 125.2, 121.3, 84.5, 43.3, 33.8, 20.5. LCMS (ESI) *m*/*z* 331.16.

#### 4.5.97 *N*-(4-*E*thylphenethyl)-5-(pyridin-2-yl)-[1,2,4]triazolo[1,5-*a*]pyrimidin-7-amine (**56**)

7-Chloro-5-(pyridin-2-yl)-[1,2,4]triazolo[1,5-*a*]pyrimidine **5k** (100 mg, 0.43 mmol) was taken in NMP (4 mL) in a 50 mL round bottom flask under N_2_. To it was added 2-(4-ethylphenyl)ethan-1-amine (70 mg, 0.52 mmol) and the reaction mixture was stirred at rt for 10 min. The reaction mixture concentrated *in vacuo* and the resulting solid taken up in EtOAc and H_2_O, the organic layer was separated and the aqueous layer extracted with EtOAc (3 × 10 mL). The organic layers combined, washed with brine, dried over Na_2_SO_4_ and the solvent removed under reduced pressure. The crude was purified by flash chromatography on silica gel (100–200 mesh) using 30% EtOAc-hexane as eluent to afford **56** as a pale yellow solid (75 mg, 50%). M.P. 154–155 °C. ^1^H NMR (400 MHz, DMSO-*d*_6_): *δ* 8.75 (d, *J* = 4.1 Hz, 1H), 8.50 (d, *J* = 5.0 Hz, 2H), 8.45 (d, *J* = 8.0 Hz, 1H), 8.00 (t, *J* = 7.7 Hz, 1H), 7.53–7.55 (m, 1H), 7.33 (s, 1H), 7.20 (d, *J* = 7.9 Hz, 2H), 7.11 (d, *J* = 7.8 Hz, 2H), 3.72–3.73 (m, 2H), 2.96–3.00 (m, 2H), 2.37 (m, 2H), 1.09 (t, *J* = 7.6 Hz, 3H). LCMS (ESI) *m*/*z* 345.39.

#### 4.5.98 *N*-(4-Fluorophenethyl)-5-(pyridin-2-yl)-[1,2,4]triazolo[1,5-*a*]pyrimidin-7-amine (**57**)

7-Chloro-5-(pyridin-2-yl)-[1,2,4]triazolo[1,5-*a*]pyrimidine **5k** (100 mg, 0.43 mmol) was taken in NMP (4 mL) in a 50 mL round bottom flask under N_2_. To it was added 2-(4-fluorophenyl)ethan-1-amine (72 mg, 0.52 mmol) and the reaction mixture was stirred at rt for 1 h. The reaction mixture concentrated *in vacuo* and the resulting solid taken up in EtOAc and H_2_O, the organic layer was separated and the aqueous layer extracted with EtOAc (3 × 10 mL). The organic layers combined, washed with brine, dried over Na_2_SO_4_ and the solvent removed under reduced pressure. The crude was purified by flash chromatography on silica gel (100–200 mesh) using 30% EtOAc-hexane as eluent to afford **57** as a white solid (69 mg, 48%). M.P. 190-192 °C. ^1^H NMR (400 MHz, DMSO-*d*_6_): *δ* 8.75 (d, *J* = 4.0 Hz, 1H), 8.51 (bs, 2H), 8.45 (d, *J* = 7.9 Hz, 1H), 7.98–8.02 (m, 1H), 7.53–7.56 (m, 1H), 7.32–7.35 (m, 3H), 7.10 (t, *J* = 8.9 Hz, 2H), 3.72–3.76 (m, 2H), 3.00–3.03 (m, 2H); ^13^C NMR (100 MHz, DMSO-*d*_6_): *δ* 160.4 (d, *J* = 240 Hz), 159.0, 155.5, 154.9, 153.8, 149.1, 148.0, 137.4, 134.8 (d, *J* = 2.9 Hz), 130.5 (d, *J* = 7.9 Hz), 125.2, 121.3, 115.0 (d, *J* = 21 Hz), 84.4, 43.2, 33.3. LCMS (ESI) *m*/*z* 335.26.

#### 4.5.99 5-(Pyridin-2-yl)-*N*-(4-(trifluoromethyl)phenethyl)-[1,2,4]triazolo[1,5-*a*]pyrimidin-7-amine (**58**)

7-Chloro-5-(pyridin-2-yl)-[1,2,4]triazolo[1,5-*a*]pyrimidine **5k** (100 mg, 0.43 mmol) was taken in NMP (4 mL) in a 50 mL round bottom flask under N_2_. To it was added 2-(4-(trifluoromethyl)phenyl)ethan-1-amine (98 mg, 0.52 mmol) and the reaction mixture was stirred at rt for 1 h. The reaction mixture concentrated *in vacuo* and the resulting solid taken up in EtOAc and H_2_O, the organic layer was separated and the aqueous layer extracted with EtOAc (3 × 20 mL). The organic layers combined, washed with brine, dried over Na_2_SO_4_ and the solvent removed under reduced pressure. The crude was purified by flash chromatography on silica gel (100–200 mesh) using 50% EtOAc-hexane as eluent to afford **58** as a pale yellow solid (80 mg, 48%). M.P. 186-188 °C. ^1^H NMR (400 MHz, DMSO-*d*_6_): *δ* 8.74 (d, *J* = 4.2 Hz, 1H), 8.56 (bs, 1H), 8.51 (s, 1H), 8.44 (d, *J* = 8.0 Hz, 1H), 8.00 (t, *J* = 7.4 Hz, 1H), 7.62–7.64 (m, 2H), 7.52–7.56 (m, 3H), 7.31 (s, 1H), 3.80–3.81 (m, 2H), 3.11–3.14 (m, 2H); ^13^C NMR (100 MHz, DMSO-*d*_6_): *δ* 159.0, 155.5, 154.9, 153.8, 149.1, 148.0, 143.8, 137.4, 129.6, 127.1 (q, *J* = 32 Hz), 125.1 (q, *J* = 5 Hz), 124.3 (q, *J* = 270 Hz), 121.3, 115.6, 84.5, 42.8, 34.0. LCMS (ESI) *m*/*z* 385.21.

#### 4.5.100 5-(Pyridin-2-yl)-*N*-(4-(trifluoromethoxy)phenethyl)-[1,2,4]triazolo[1,5-*a*]pyrimidin-7-amine (**59**)

7-Chloro-5-(pyridin-2-yl)-[1,2,4]triazolo[1,5-*a*]pyrimidine **5k** (100 mg, 0.43 mmol) was taken in NMP (4 mL) in a 50 mL round bottom flask under N_2_. To it was added 2-(4-(trifluoromethoxy)phenyl)ethan-1-amine (107 mg, 0.52 mmol) and the reaction mixture was stirred at rt for 0.5 h. The reaction mixture concentrated *in vacuo* and the resulting solid taken up in EtOAc and H_2_O, the organic layer was separated and the aqueous layer extracted with EtOAc (3 × 20 mL). The organic layers combined, washed with brine, dried over Na_2_SO_4_ and the solvent removed under reduced pressure. The crude was purified by flash chromatography on silica gel (100–200 mesh) using 32% EtOAc-hexane as eluent to afford **59** as a white solid (45 mg, 48%). M.P. 163-165 °C. ^1^H NMR (400 MHz, DMSO-*d*_6_): *δ* 8.74 (d, *J* = 4.3 Hz, 1H), 8.55 (bs, 1H), 8.54 (s, 1H), 8.45 (d, *J* = 8.0 Hz, 1H), 8.01 (t, *J* = 7.6 Hz, 1H), 7.53–7.56 (m, 1H), 7.43 (d, *J* = 8.5 Hz, 2H), 7.33 (s, 1H), 7.26 (d, *J* = 7.9 Hz, 2H), 3.75–3.80 (m, 2H), 3.04–3.08 (m, 2H); ^13^C NMR (100 MHz, DMSO-*d*_6_): *δ* 159.0, 155.5, 154.9, 153.8, 149.2, 148.1, 146.9, 138.3, 137.4, 130.6, 125.2, 121.4, 120.9, 120.0 (q, *J* = 260 Hz), 84.5, 43.0, 33.5. LCMS (ESI) *m*/*z* 401.10.

#### 4.5.101 *N*-(4-Chlorophenethyl)-5-(pyridin-2-yl)-[1,2,4]triazolo[1,5-*a*]pyrimidin-7-amine (**60**)

7-Chloro-5-(pyridin-2-yl)-[1,2,4]triazolo[1,5-*a*]pyrimidine **5k** (120 mg, 0.52 mmol) was taken in NMP (3 mL) in a 50 mL round bottom flask under N_2_. To it was added 2-(4-chlorophenyl)ethan-1-amine (96 mg, 0.62 mmol) and the reaction mixture was stirred at rt for 30 min. The reaction mixture concentrated *in vacuo* and the resulting solid taken up in EtOAc and H_2_O, the organic layer was separated and the aqueous layer extracted with EtOAc (3 × 20 mL). The organic layers combined, washed with brine, dried over Na_2_SO_4_ and the solvent removed under reduced pressure. The crude was purified by flash chromatography on silica gel (100–200 mesh) using 30% EtOAc-hexane as eluent to afford **60** as a white solid (60 mg, 33%). M.P. 194–195 °C. ^1^H NMR (400 MHz, DMSO-*d*_6_): *δ* 8.75 (d, *J* = 3.8 Hz, 1H), 8.51 (m, 2H), 8.45 (d, *J* = 7.5 Hz, 1H), 8.08 (t, *J* = 7.2 Hz, 1H), 7.53–7.56 (m,1H), 7.33 (m, 5H), 3.74–3.76 (m, 2H), 3.00–3.04 (m, 2H); ^13^C NMR (100 MHz, DMSO-*d*_6_): *δ* 159.5, 156.0, 155.4, 154.3, 149.6, 148.5, 138.3, 137.9, 131.5, 131.2, 128.7, 125.7, 121.8, 84.9, 43.5, 33.9. LCMS (ESI) *m*/*z* 351.10.

#### 4.5.102 *N*-(4-Methoxyphenethyl)-*N*-methyl-5-phenyl-[1,2,4]triazolo[1,5-*a*]pyrimidin-7-amine (**61**)

Sodium hydride (25 mg, 1.1 mmmol) was taken in anhydrous DMF (5 mL) in a 50 mL round bottom flask under N_2_ and cooled it down to 0 °C. To it was added a solution of *N*-(4-methoxyphenethyl)-5-phenyl-[1,2,4]triazolo[1,5-*a*]pyrimidin-7-amine **1** (250 g, 0.72 mmol) in DMF (1 mL). It was kept stirred at rt for 30 min. Methyl iodide (120 mg, 0.86 mmol) was then added to it and the reaction mixture was stirred at rt for 2 h. Ice-cooled water was added dropwise to quench the reaction. It was extracted with EtOAc (3 × 20 mL). The combined organic layer was dried over anhydrous Na_2_SO_4_ and concentrated under reduced pressure. The crude was further purified by flash chromatography on silica gel (60–120 mesh) using 50% hexane-EtOAc as eluent to afford **61** as a pale yellow solid (180 mg, 69%). M.P. 170-171 °C. ^1^H NMR (400 MHz, DMSO-*d*_6_): *δ* 8.47 (s, 1H), 8.16–8.18 (m, 2H), 7.53–7.54 (m, 3H), 7.16 (d, *J* = 8.5 Hz, 2H), 6.75 (d, *J* = 8.6 Hz, 3H), 4.32–4.35 (m, 2H), 3.65 (s, 3H), 3.34 (s, 3H), 2.92–2.96 (m, 2H); ^13^C NMR (100 MHz, DMSO-*d*_6_): *δ* 159.7, 157.8, 157.2, 154.2, 149.9, 137.4, 130.3, 130.2, 129.8, 128.6, 127.3, 113.6, 89.8, 54.9, 54.1, 40.1, 33.3. LCMS (ESI) *m*/*z* 360.22.

#### 4.5.103 7-(3-(4-Methoxyphenyl)pyrrolidin-1-yl)-5-(pyridin-2-yl)-[1,2,4]triazolo[1,5-*a*]pyrimidine (**62**)

7-Chloro-5-(pyridin-2-yl)-[1,2,4]triazolo[1,5-*a*]pyrimidine **5k** (100 mg, 0.43 mmol) was taken in NMP (2 mL) in a 50 mL round bottom flask under N_2_. To it was added 3-(4-methoxyphenyl)pyrrolidine (92 mg, 0.52 mmol) and the reaction mixture was heated at 75 °C for 2 h. The reaction mixture concentrated *in vacuo* and the resulting solid taken up in EtOAc and H_2_O, the organic layer was separated and the aqueous layer extracted with EtOAc (3 × 20 mL). The organic layers combined, washed with brine, dried over Na_2_SO_4_ and the solvent removed under reduced pressure. The crude was purified by flash chromatography on silica gel (100–200 mesh) using 3% MeOH-DCM as eluent to afford **62** as a light yellow solid (46 mg, 29%). M.P. 222–223 °C. ^1^H NMR (400 MHz, CDCl_3_): *δ* 8.64–8.66 (m, 2H), 8.26 (s, 1H), 7.86 (t, *J* = 7.8 Hz, 1H), 7.36–7.39 (m, 1H), 7.23–7.26 (m, 3H), 6.91 (d, *J* = 8.5 Hz, 2H), 4.60 (m, 1H), 4.39 (m, 1H), 4.16 (m, 1H), 4.04 (m, 1H), 3.81 (s, 3H), 3.48–3.56 (m, 1H), 2.45–2.50 (m, 1H), 2.16–2.26 (m, 1H); ^13^C NMR (100 MHz, CDCl_3_): *δ* 160.0, 158.9, 158.0, 154.6, 154.5, 148.9, 148.3, 137.2, 132.0, 128.2, 125.0, 122.4, 114.3, 88.2, 55.5, 51.0, 43.3, 32.1, 22.8. LCMS (ESI) *m*/*z* 373.19.

#### 4.5.104 7-(3-(4-Methoxyphenyl)piperidin-1-yl)-5-(pyridin-2-yl)-[1,2,4]triazolo[1,5-*a*]pyrimidine (**63**)

(a) 1-Bromo-4-methoxybenzene (500 mg, 2.7 mmol) was taken in DMF (8 mL) in a 50 mL round bottom flask under N_2_. To it were sequentially added pyridin-3-ylboronic acid (394 mg, 3.2 mmol), Pd(OAc)_2_ (60 mg, 0.27 mmol), dppf (149 mg, 0.27 mmol), CuCl (27 mg, 0.27 mmol) and Cs_2_CO_3_ (1.7 g, 5.4 mmol). The reaction mixture was heated at 110 °C for 2 h. It was then filtered through sintered funnel with a pad of Celite, washed with EtOAc (40 mL). The filtrate was then poured into ice water (40 g) and extracted with EtOAc (3 × 50 mL). The combined organic layer was dried over anhydrous Na_2_SO_4_ and concentrated under reduced pressure. The crude was purified by flash chromatography on silica gel (100–200 mesh) using 15% EtOAc-hexane as eluent to afford 3-(4-methoxyphenyl)pyridine as a light brown liquid (325 mg, 66%). ^1^H NMR (400 MHz, DMSO-*d*_6_): *δ* 8.85 (s, 1H), 8.50–8.51 (m, 1H), 8.02 (d, *J* = 7.9 Hz, 1H), 7.68 (d, *J* = 8.6 Hz, 2H), 7.43–7.46 (m, 1H), 7.06 (d, *J* = 8.7 Hz, 2H), 3.81 (s, 3H). (b) A par flask was charged with 3-(4-methoxyphenyl)pyridine **49** (140 mg, 0.75 mmole) and MeOH (5 mL) followed by addition of HCl () and PtO_2_ (205 mg, 0.9 mmol). The flask was evacuated under vacuum and then purged with hydrogen. The reaction was stirred under hydrogen atmosphere (20 psi) for 6 h. It was then filtered through sintered funnel with a pad of Celite, washed with MeOH (20 mL) and concentrated under reduced pressure to afford 3-(4-methoxyphenyl)piperidine as a white solid (100 mg, 69%) that was used as such for the next step without any further purification. LCMS(ESI) *m*/*z* 192.10 (M+H)^+^, 97.34% (purity). (c) 7-Chloro-5-(pyridin-2-yl)-[1,2,4]triazolo[1,5-*a*]pyrimidine **35** (100 mg, 0.43 mmol) was taken in NMP (3 mL) in a 50 mL round bottom flask under N_2_. To it was added 3-(4-methoxyphenyl)piperidine (99 mg, 0.52 mmol) and the reaction mixture was stirred at rt for 30 min. The reaction mixture concentrated *in vacuo* and the resulting solid taken up in EtOAc and H_2_O, the organic layer was separated and the aqueous layer extracted with EtOAc (3 × 20 mL). The organic layers combined, washed with brine, dried over Na_2_SO_4_ and the solvent removed under reduced pressure. The crude was purified by flash chromatography on silica gel (100–200 mesh) using 34% EtOAc-hexane as eluent to afford **63** as a semi-solid (50 mg, 30%). ^1^H NMR (400 MHz, DMSO-*d*_6_): *δ* 8.75 (d, *J* = 4.0 Hz, 1H), 8.54 (s, 1H), 8.48 (d, *J* = 7.9 Hz, 1H), 8.01–8.05 (m, 1H), 7.55–7.58 (m, 2H), 7.28 (d, *J* = 8.6 Hz, 2H), 6.92 (d, *J* = 8.6 Hz, 2H), 4.73–4.76 (m, 1H), 4.64–4.68 (m, 1H), 3.74 (s, 3H), 3.25–3.28 (m, 2H), 2.92–2.94 (m, 1H), 1.95–1.98 (m, 2H), 1.81–1.89 (m, 2H); ^13^C NMR (100 MHz, DMSO-*d*_6_): *δ* 158.8, 158.0, 157.1, 154.6, 153.4, 150.4, 149.3, 137.6, 134.9, 128.1, 125.5, 121.4, 113.9, 90.8, 55.0, 54.4, 48.4, 31.2, 24.8. LCMS (ESI) *m*/*z* 387.23.

#### 4.5.105 2-(4-Methoxyphenyl)-4-(5-(pyridin-2-yl)-[1,2,4]triazolo[1,5-*a*]pyrimidin-7-yl)morpholine (**64**)

(a) 2-Bromo-1-(4-methoxyphenyl)ethan-1-one (4.0 g, 17.5 mmmol) was taken in dry THF (45 mL) in a 100 mL round bottom flask under N_2_ and cooled it down to 0 °C. To it was added a solution of 2-(benzylamino)ethan-1-ol (3.7 g, 24.4 mmol) in THF (5 mL). The reaction mixture was heated at 50 °C for 12 h. Ice-cooled water was added dropwise to quench the reaction. It was extracted with EtOAc (3 × 75 mL). The combined organic layer was dried over anhydrous Na_2_SO_4_ and concentrated under reduced pressure. The crude was further purified by flash chromatography on silica gel (60–120 mesh) using 15% hexane-EtOAc as eluent to afford 2-(benzyl(2-hydroxyethyl)amino)-1-(4-methoxyphenyl)ethan-1-one as a brown solid (2.5 g, 48%). LCMS(ESI) *m*/*z* 300.09 [M+H^+^]; 52% (purity). (b) 2-(Benzyl(2-hydroxyethyl)amino)-1-(4-methoxyphenyl)ethan-1-one (2.5 g, 8.3 mmmol) was taken in MeOH (20 mL) in a 100 mL round bottom flask under N_2_ and cooled it down to 0 °C. To it was added sodiumboro hydride (3.7 g, 24.4 mmol) in portion-wise. The reaction mixture was stirred at rt for 3 h. The reaction mixture was evaporated to dryness. Ice-cooled water was added dropwise to quench the reaction. It was extracted with EtOAc (3 × 100 mL). The combined organic layer was dried over anhydrous Na_2_SO_4_ and concentrated under reduced pressure. This afforded 2-(benzyl(2-hydroxyethyl)amino)-1-(4-methoxyphenyl)ethan-1-ol as a brown solid (1.2 g, 48%). This was then used in the next step without any further purification. LCMS(ESI) *m*/*z* 302.21 [M+H^+^]; 42% (purity). (c) 2-(Benzyl(2-hydroxyethyl)amino)-1-(4-methoxyphenyl)ethan-1-ol (1.2 g, 3.9 mmmol) was taken in HCl:H_2_O mixture (10 mL, 60% HCl in H_2_O) in a 50 mL round bottom flask fitted with a condenser. The reaction mixture was heated at 110 °C for 12 h. The reaction mixture was evaporated to dryness under reduced pressure. This was triturated with Et_2_O which afforded 4-benzyl-2-(4-methoxyphenyl)morpholine as a brown semi-solid (800 mg, 71%). This was then used in the next step without any further purification. LCMS(ESI) *m*/*z* 284.10 [M+H^+^]; 98.71% (purity). (d) A par flask was charged with 4-benzyl-2-(4-methoxyphenyl)morpholine (800 mg, 2.8 mmmol) and MeOH (20 mL) followed by the addition of Pd(OH)_2_ (393 mg, 2.8 mmol). The flask was evacuated under vacuum and then purged with hydrogen. The reaction was stirred under hydrogen atmosphere (20 psi) for 4 h. It was then filtered through sintered funnel with a pad of Celite, washed with MeOH (20 mL) and concentrated under reduced pressure to afford 2-(4-methoxyphenyl)morpholine as a white solid (500 mg, 92%) that was used as such for the next step without any further purification. LCMS(ESI) *m*/*z* 194.28 (M+H)^+^; 85% (purity). (e) 7-Chloro-2-methyl-5-(pyridin-2-yl)pyrazolo[1,5-*a*]pyrimidine (200 mg, 0.86 mmol) was taken in NMP (6 mL) in a 50 mL round bottom flask under N_2_. To it was added 2-(4-methoxyphenyl)morpholine (200 mg, 1.04 mmol) and the reaction mixture was stirred at rt for 1 h. The reaction mixture concentrated *in vacuo* and the resulting solid taken up in EtOAc and H_2_O, the organic layer was separated and the aqueous layer extracted with EtOAc (3 × 20 mL). The organic layers combined, washed with brine, dried over Na_2_SO_4_ and the solvent removed under reduced pressure. The crude was purified by flash chromatography on silica gel (100–200 mesh) using 40% EtOAc-hexane as eluent to afford **64** as a white solid (130 mg, 39%). ^1^H NMR (400 MHz, DMSO-*d*_6_): *δ* 8.75 (d, *J* = 4.4 Hz, 1H), 8.59 (s, 1H), 8.49 (d, *J* = 7.9 Hz, 1H), 8.01–8.06 (m, 1H), 7.56–7.60 (m, 2H), 7.40 (d, *J* = 8.6 Hz, 2H), 6.97 (d, *J* = 8.6 Hz, 2H), 4.70–4.75 (m, 2H), 4.58–4.70 (m, 1H), 4.17–4.19 (m, 1H), 3.92–3.97 (m, 1H), 3.76 (s, 3H), 3.39–3.46 (m, 1H), 3.21–3.27 (m, 1H); ^13^C NMR (100 MHz, DMSO-*d*_6_): *δ* 159.1, 159.0, 156.9, 154.8, 153.3, 150.5, 149.3, 137.6, 131.2, 127.6, 125.6, 121.4, 113.7, 90.9, 76.3, 65.6, 55.1, 53.3, 47.2. LCMS (ESI) *m*/*z* 389.16.

#### 4.5.106 *N*-(4-Methoxyphenethyl)-2-methyl-5-(pyridin-2-yl)-[1,2,4]triazolo[1,5-*a*]pyrimidin-7-amine (**67**)

(a) Ethyl 3-oxo-3-(pyridin-2-yl)propanoate (400 mg, 2.07 mmol) was taken in AcOH (10 mL) in a 50 mL round bottom flask under N_2_. To it was added 5-methyl-4*H*-1,2,4-triazol-3-amine (334 mg, 2.49 mmol). The reaction mixture was heated at 115 °C for 12 h. The reaction mixture was then evaporated to dryness using toluene as an azeotropic solvent and triturated with diethyl ether. This was finally dried under high vaccum which affored **66a** as a brown solid (350 mg, 75%). This was then used in the next step without any further purification. LCMS(ESI) *m*/*z* 226.03 [M−H^+^]; 56% (purity). (b) To a solution of 2-methyl-5-(pyridin-2-yl)pyrazolo[1,5-*a*]pyrimidin-7-ol **66a** (350 mg, 1.5 mmol) was added POCl_3_ (6 mL, 60 mmol) at 0 °C. The reaction mixture was then heated at 100 °C and monitored by TLC analysis (Hexane/EtOAc = 1:1). Upon completion, the reaction mixture was concentrated using toluene as an azeotropic solvent and quenched with ice cooled saturated NaHCO_3_ solution (20 mL) to pH 8. It was extracted with EtOAc (3 × 50 mL). The combined organic layer was dried over anhydrous Na_2_SO_4_ and concentrated under reduced pressure. The crude was further purified by flash chromatography on silica gel (60–120 mesh) using 15% EtOAc-hexane as eluent to afford 7-chloro-2-methyl-5-(pyridin-2-yl)pyrazolo[1,5-*a*]pyrimidine as a brown solid (125 mg, 33%). LCMS(ESI) *m*/*z* 246.20 [M+H^+^]; 49% (purity). (c) 7-Chloro-2-methyl-5-(pyridin-2-yl)pyrazolo[1,5-*a*]pyrimidine (100 mg, 0.41 mmol) was taken in NMP (2 mL) in a 50 mL round bottom flask under N_2_. To it was added 2-(4-methoxyphenyl)ethan-1-amine **5a** (74 mg, 0.49 mmol) and the reaction mixture was stirred at rt for 30 min. The reaction mixture concentrated *in vacuo* and the resulting solid taken up in EtOAc and H_2_O, the organic layer was separated and the aqueous layer extracted with EtOAc (3 × 20 mL). The organic layers combined, washed with brine, dried over Na_2_SO_4_ and the solvent removed under reduced pressure. The crude was purified by flash chromatography on silica gel (100–200 mesh) using 35% EtOAc-hexane as eluent to afford **67** as a white solid (80 mg, 54%). M.P. 139-141 °C. ^1^H NMR (400 MHz, DMSO-*d*_6_): *δ* 8.73–8.74 (m, 1H), 8.39–8.42 (m, 2H), 7.97–8.01 (m, 1H), 7.51–7.55 (m, 1H), 7.27 (s, 1H), 7.20 (d, *J* = 8.6 Hz, 2H), 6.84 (d, *J* = 8.6 Hz, 2H), 3.65–3.70 (m, 5H), 2.92–2.95 (m, 2H), 2.50 (s, 3H); ^13^C NMR (100 MHz, DMSO-*d*_6_): *δ* 164.1, 158.4, 157.8, 155.9, 153.9, 149.1, 147.5, 137.3, 130.5, 129.7, 125.1, 121.2, 113.8, 84.2, 54.9, 43.4, 33.4, 14.8. LCMS (ESI) *m*/*z* 359.06.

#### 4.5.107 *N*-(4-Methoxyphenethyl)-2-phenyl-5-(pyridin-2-yl)-[1,2,4]triazolo[1,5-*a*]pyrimidin-7-amine (**68**)

(a) Ethyl 3-oxo-3-(pyridin-2-yl)propanoate (500 mg, 2.6 mmol) was taken in AcOH (50 mL) in a 250 mL round bottom flask under N_2_. To it was added 3-phenyl-1*H*-1,2,4-triazol-5-amine (497 mg, 3.1 mmol). The reaction mixture was heated at 115 °C for 12 h. The reaction mixture was then evaporated to dryness using toluene as an azeotropic solvent and triturated with diethyl ether. This was finally dried under high vacuum which afforded **66b** as a deep brown solid (300 mg, 40%). This was then used in the next step without any further purification. LCMS(ESI) *m*/*z* 288.02 [M−H^+^]; 57% (purity). (b) To a solution of 2-phenyl-5-(pyridin-2-yl)-[1,2,4]triazolo[1,5-*a*]pyrimidin-7-ol **66b** (300 mg, 1.04 mmol) was added POCl_3_ (2 mL, 21 mmol) at 0 °C. The reaction mixture was then heated at 100 °C and monitored by TLC analysis (Hexane/EtOAc = 3:7). Upon completion, the reaction mixture was concentrated using toluene as an azeotropic solvent and quenched with ice cooled saturated NaHCO_3_ solution (20 mL) to pH 8. It was extracted with EtOAc (3 × 50 mL). The combined organic layer was dried over anhydrous Na_2_SO_4_ and concentrated under reduced pressure. The crude was further purified by flash chromatography on silica gel (60–120 mesh) using 17% hexane-EtOAc as eluent to afford 7-chloro-2-phenyl-5-(pyridin-2-yl)-[1,2,4]triazolo[1,5-*a*]pyrimidine as a brown solid (150 mg, 47%).LCMS(ESI) *m*/*z* 307.99 [M+H^+^]; 87.88% (purity). (c) 7-Chloro-2-phenyl-5-(pyridin-2-yl)-[1,2,4]triazolo[1,5-*a*]pyrimidine (125 mg, 0.41 mmol) was taken in NMP (2 mL) in a 50 mL round bottom flask under N_2_. To it was added 2-(4-methoxyphenyl)ethan-1-amine (74 mg, 0.49 mmol) and the reaction mixture was stirred at rt for 30 min. The reaction mixture concentrated *in vacuo* and the resulting solid taken up in EtOAc and H_2_O, the organic layer was separated and the aqueous layer extracted with EtOAc (3 × 20 mL). The organic layers combined, washed with brine, dried over Na_2_SO_4_ and the solvent removed under reduced pressure. The crude was purified by flash chromatography on silica gel (100–200 mesh) using 50% EtOAc-hexane as eluent to afford **68** as a white solid (65 mg, 38%). M.P. 146–148 °C. ^1^H NMR (400 MHz, DMSO-*d*_6_): *δ* 8.76 (d, *J* = 4.0 Hz, 1H), 8.47 (d, *J* = 7.8 Hz, 2H), 8.25–8.27 (m, 2H), 8.00–8.05 (m, 1H), 7.54–7.59 (m, 4H), 7.36 (s, 1H), 7.24 (d, *J* = 8.5 Hz, 2H), 6.86 (d, *J* = 8.5 Hz, 2H), 3.73–3.75 (m, 2H), 3.67 (s, 3H), 2.97–3.01 (m, 2H); ^13^C NMR (100 MHz, DMSO-*d*_6_): *δ* 163.6, 158.8, 157.8, 156.3, 153.9, 149.2, 147.8, 137.4, 130.8, 130.5, 130.3, 129.7, 128.8, 126.8, 125.3, 121.3, 113.9, 84.8, 54.9, 43.6, 33.5. LCMS (ESI) *m*/*z* 423.20.

#### 4.5.108 *N*-(4-Methoxyphenethyl)-5-(pyridin-2-yl)pyrazolo[1,5-*a*]pyrimidin-7-amine (**69**)

(a) Ethyl 3-oxo-3-(pyridin-2-yl)propanoate (500 mg, 2.6 mmol) was taken in AcOH (6 mL) in a 50 mL round bottom flask under N_2_. To it was added 1*H*-pyrazol-3-amine (258 mg, 3.1 mmol). The reaction mixture was heated at 120 °C for 14 h. The reaction mixture was then evaporated to dryness using toluene as an azeotropic solvent and triturated with diethyl ether. This was finally dried under high vaccum which affored **66c** as a colourless solid (420 mg, 77%). This was then used in the next step without any further purification. MS (ESI) *m*/*z* 213.08 [M+H^+^]. (b) To a solution of 5-(pyridin-2-yl)pyrazolo[1,5-*a*]pyrimidin-7-ol **66c** (400 mg, 1.8 mmol) was added POCl_3_ (6 mL, 66.5 mmol) at 0 °C. The reaction mixture was then heated at 100 °C and monitored by TLC analysis (Hexane/EtOAc = 3:7). Upon completion, the reaction mixture was concentrated using toluene as an azeotropic solvent and quenched with ice cooled saturated NaHCO_3_ solution (20 mL) to pH 8. It was extracted with EtOAc (3 × 50 mL). The combined organic layer was dried over anhydrous Na_2_SO_4_ and concentrated under reduced pressure. The crude was further purified by flash chromatography on silica gel (60–120 mesh) using 5% MeOH-DCM as eluent to afford 7-chloro-5-(pyridin-2-yl)pyrazolo[1,5-*a*]pyrimidine as a brown liquid (320 mg, 74%). LCMS(ESI) *m*/*z* 231.06 [M+H^+^]; 75.59% (purity). (c) 7-Chloro-5-(pyridin-2-yl)pyrazolo[1,5-*a*]pyrimidine (100 mg, 0.43 mmol) was taken in NMP (2 mL) in a 50 mL round bottom flask under N_2_. To it was added 2-(4-methoxyphenyl)ethan-1-amine (79 mg, 0.52 mmol) and the reaction mixture was stirred at rt for 30 min. The reaction mixture concentrated *in vacuo* and the resulting solid taken up in EtOAc and H_2_O, the organic layer was separated and the aqueous layer extracted with EtOAc (3 × 20 mL). The organic layers combined, washed with brine, dried over Na_2_SO_4_ and the solvent removed under reduced pressure. The crude was purified by flash chromatography on silica gel (100–200 mesh) using 35% EtOAc-hexane as eluent to afford **69** as a light yellow solid (30 mg, 20%). M.P. 116–117 °C. ^1^H NMR (400 MHz, DMSO-*d*_6_): *δ* 8.72–8.73 (m, 1H), 8.42 (d, *J* = 7.9 Hz, 1H), 8.13 (d, *J* = 2.2 Hz, 1H), 8.01–8.05 (m, 1H), 7.93–7.98 (m, 1H), 7.48–7.51 (m, 1H), 7.23 (d, *J* = 8.6 Hz, 2H), 7.15 (s, 1H), 6.86 (d, *J* = 8.6 Hz, 2H), 6.53 (d, *J* = 2.2 Hz, 1H), 3.65–3.71 (m, 5H), 2.95–2.99 (m, 2H); ^13^C NMR (100 MHz, DMSO-*d*_6_): *δ* 157.8, 154.8, 154.6, 149.0, 148.6, 147.0, 143.9, 137.1, 130.6, 129.6, 124.6, 120.9, 113.8, 95.3, 81.2, 54.9, 43.2, 33.4. LCMS (ESI) *m*/*z* 346.18.

#### 4.5.109 *N*-(4-Methoxyphenethyl)-7-(pyridin-2-yl)imidazo[1,2-*a*]pyrimidin-5-amine (**70**)

(a) Ethyl 3-oxo-3-(pyridin-2-yl)propanoate (2.0 g, 10.3 mmol) was taken in AcOH (20 mL) in a 100 mL round bottom flask under N_2_. To it was added 1*H*-imidazol-2-amine (1.03 g, 12.4 mmol). The reaction mixture was heated at 120 °C for 12 h. The reaction mixture was then evaporated to dryness using toluene as an azeotropic solvent and triturated with diethyl ether. This was finally dried under high vacuum which afforded **66d** as a deep brown solid (1.0 g, 45%). This was then used in the next step without any further purification. LCMS(ESI) *m*/*z* 212.98 [M+H^+^]; 52% (purity). (b) To a solution of 7-(pyridin-2-yl)imidazo[1,2-*a*]pyrimidin-5-ol **66d** (700 mg, 3.3 mmol) was added POCl_3_ (10 mL, 105.6 mmol) at 0 °C. The reaction mixture was then heated at 100 °C and monitored by TLC analysis (Hexane/EtOAc = 3:7). Upon completion, the reaction mixture was concentrated using toluene as an azeotropic solvent and quenched with ice cooled saturated NaHCO_3_ solution (20 mL) to pH 8. It was extracted with EtOAc (3 × 50 mL). The combined organic layer was dried over anhydrous Na_2_SO_4_ and concentrated under reduced pressure. The crude was further purified by flash chromatography on silica gel (60–120 mesh) using 5% MeOH-DCM as eluent to afford 5-chloro-7-(pyridin-2-yl)imidazo[1,2-*a*]pyrimidine as a brown liquid (350 mg, 46%). LCMS(ESI) *m*/*z* 231.03 [M+H^+^]; 92.15% (purity). (c) 5-Chloro-7-(pyridin-2-yl)imidazo[1,2-*a*]pyrimidine (100 mg, 0.43 mmol) was taken in NMP (2 mL) in a 50 mL round bottom flask under N_2_. To it was added 2-(4-methoxyphenyl)ethan-1-amine **5a** (79 mg, 0.52 mmol) and the reaction mixture was stirred at rt for 30 min. The reaction mixture concentrated *in vacuo* and the resulting solid taken up in EtOAc and H_2_O, the organic layer was separated and the aqueous layer extracted with EtOAc (3 × 20 mL). The organic layers combined, washed with brine, dried over Na_2_SO_4_ and the solvent removed under reduced pressure. The crude was purified by flash chromatography on silica gel (100–200 mesh) using 2% MeOH-DCM as eluent to afford **70** as a light yellow solid (32 mg, 21%). M.P. 234–235 °C. ^1^H NMR (400 MHz, DMSO-*d*_6_): *δ* 8.71–8.72 (m, 1H), 8.44 (d, *J* = 7.9 Hz, 1H), 7.95–7.99 (m, 2H), 7.90–7.92 (m, 1H), 7.62 (d, *J* = 1.4 Hz, 1H), 7.48–7.51 (m, 1H), 7.23 (d, *J* = 8.5 Hz, 2H), 7.15 (d, *J* = 6.5 Hz, 1H), 6.87 (d, *J* = 8.6 Hz, 2H), 3.67 (s, 3H), 3.62–3.64 (m, 2H), 2.93–2.99 (m, 2H); ^13^C NMR (100 MHz, DMSO-*d*_6_): *δ* 157.9, 155.6, 154.7, 149.8, 149.0, 147.4, 137.2, 133.9, 130.7, 129.7, 124.7, 120.8, 113.8, 106.2, 81.4, 54.9, 43.9, 33.1. LCMS (ESI) *m*/*z* 346.19.
